# SRC-mediated phosphorylation of UBC9 regulates inflammatory and metabolic signaling in alcohol-associated liver disease

**DOI:** 10.1126/sciadv.aec0138

**Published:** 2026-04-10

**Authors:** Swati Chandla, Youngyi Lim, Andrea Floris, Michael Mazarei, Xi Yang, Takashi Tsuchiya, Manisha Dagar, Alfonso Darmawan, Monica Justo, Ramachandran Murali, Alexandra Gangi, Ivan Tomasi, Nirmala Mavila, Komal Ramani, Maria Lauda Tomasi

**Affiliations:** ^1^ Karsh Division of Gastroenterology and Hepatology, Department of Medicine, Cedars-Sinai Medical Center, Los Angeles, CA 90048 USA.; ^2^ Department of Surgery, Cedars-Sinai Medical Center, Los Angeles, CA 90048 USA.; ^3^ Department of Biomedical Sciences, Cedars-Sinai Medical Center, Los Angeles, CA 90048 USA.; ^4^ Department of General Surgery, Guys and St Thomas Hospital, London SE1 7EH, UK.

## Abstract

Alcohol-associated liver disease (ALD) remains a major public health challenge with limited treatment options. NF-κB–driven inflammation in Kupffer cells (KCs) plays a central role in ALD, but the upstream regulators remain poorly understood. Here, we identify the tyrosine kinase SRC as a key mediator of ALD. Chronic ethanol exposure activates SRC in KCs, which directly phosphorylates ubiquitin-conjugating enzyme 9 (UBC9), the only E2 SUMO enzyme, at tyrosine-68 (Y68). This modification enhances NF-κB signaling and increases proinflammatory cytokines (TNF-α, IL-6, and IL-1β). These cytokines then promote hepatic lipogenesis through SREBP1c- and CEBPβ-dependent induction of FASN and ACC. Inhibition of UBC9 phosphorylation by gene editing or SRC inhibitor reduces NF-κB–dependent inflammation and lessens ethanol-induced liver injury in mouse models. These findings uncover a previously unrecognized SRC–UBC9–NF-κB axis that drives inflammation in ALD and highlight it as a potential therapeutic target in liver disease.

## Teaser

The SRC-UBC9 signaling axis regulates alcohol-associated liver disease progression and represents a potential therapeutic target.

## INTRODUCTION

Alcohol-associated liver disease (ALD) is characterized by a spectrum of progressive liver damage, ranging from steatosis to cirrhosis and hepatocellular carcinoma. Despite the global awareness of alcohol’s detrimental effects, alcohol consumption continues to rise, contributing to the growing incidence of ALD worldwide (*1*, *2*). Furthermore, the lack of effective treatments is one of the pivotal contributing factors for ALD (*2*, *3*). Chronic alcohol consumption is known to activate Kupffer cells (KCs) via increased circulating lipopolysaccharide (LPS) levels (*4*) and promote LPS-mediated nuclear factor κB (NF-κB) activation, leading to the production of proinflammatory cytokines such as tumor necrosis factor–α (TNF-α), interleukin-6 (IL-6), and IL-1β (*5*, *6*)*.* These cytokines have been shown to regulate lipogenic programs in hepatocytes by activating sterol regulatory element–binding protein 1c (SREBP1c) and CCAAT/enhancer-binding protein beta (CEBPβ) (*7*, *8*). A critical assignment of this inflammatory response is the activation of the NF-κB pathway, which drives cytokine production, immune cell recruitment, and liver injury (*9*). While the effects of NF-κB activation have been extensively studied, the upstream signaling events that initiate and sustain NF-κB activation in ALD remain incompletely understood. Therefore, a deeper understanding of the molecular and biochemical mechanisms underlying ALD is crucial for developing targeted therapeutic strategies.

Ubiquitin-conjugating enzyme 9 (UBC9) is the sole E2 small ubiquitin-like modifier (SUMO)–conjugating enzyme responsible for all protein SUMOylation in mammalian cells, a posttranslational modification process. UBC9 controls the conjugation of SUMO proteins to target proteins, modulating their stability, intracellular localization, and transcriptional activity (*10*, *11*). We previously reported cyclin-dependent kinase 1–mediated phosphorylation of UBC9 in cancer cells (*12*). We also reported that alcohol exposure in animal models leads to elevated UBC9 expression in total liver (*12*). UBC9 has also been implicated in inflammatory signaling, particularly through the SUMOylation of the inhibitor of NF-κB α (IκBα), with SUMO-2/3 modification promoting IκBα degradation and SUMO-1 stabilizing IκBα (*13*, *14*). We previously demonstrated that total UBC9 deficiency in LPS-activated KCs exacerbates NF-κB–driven inflammatory cytokine production, while total UBC9 overexpression suppresses this response (*10*). We further discovered that LPS-induced phosphorylation of UBC9 enhances its interaction with IκBα protein and speculated that UBC9 phosphorylation might promote NF-κB signaling by quenching the ability of IκBα to control NF-κB (*10*). However, the molecular mechanisms governing UBC9 regulation in ALD are unknown.

In this study, we uncover SRC, a nonreceptor tyrosine kinase, as a key upstream regulator of UBC9 and their interaction as a driver of inflammation in ALD. SRC integrates signals from diverse extracellular stimuli, including cytokines, growth factors, integrins, and cellular stress, and modulates key processes such as proliferation, migration, cytoskeletal dynamics, and immune responses (*15*). SRC activity is tightly regulated by autophosphorylation at tyrosine 416 (Y416) that induces an active conformation (*16*, *17*). Emerging data suggest that SRC may serve as a signaling linker between inflammatory stress and posttranslational regulation of key transcriptional pathways. In macrophages, SRC modulates the activity of signal transducers and activators of transcription (STATs), mitogen-activated protein kinases, and NF-κB (*18*), but whether it also governs SUMO-related modifications remains unknown. Moreover, SRC expression has been studied in nonalcoholic steatohepatitis, and it has been reported to activate focal adhesion kinase/phosphatidylinositol 3-kinase/Akt pathway in vitro (*19*). However, the underlying molecular mechanism regulating SRC-UBC9 axis involved in the progression of ALD is largely unexplored in vitro as well as in vivo.

In this study, we investigate the potential functions of SRC as an upstream modulator of UBC9 in KCs, linking tyrosine kinase signaling to SUMOylation-dependent activation of NF-κB. By elucidating this pathway, we aim to illustrate a previously unknown signaling axis that connects ethanol-induced stress to KC activation in the liver and identify therapeutic targets for ALD.

## RESULTS

### Phosphorylation of UBC9 at Y68 is induced in KCs upon alcohol exposure

**Fig. 1.**
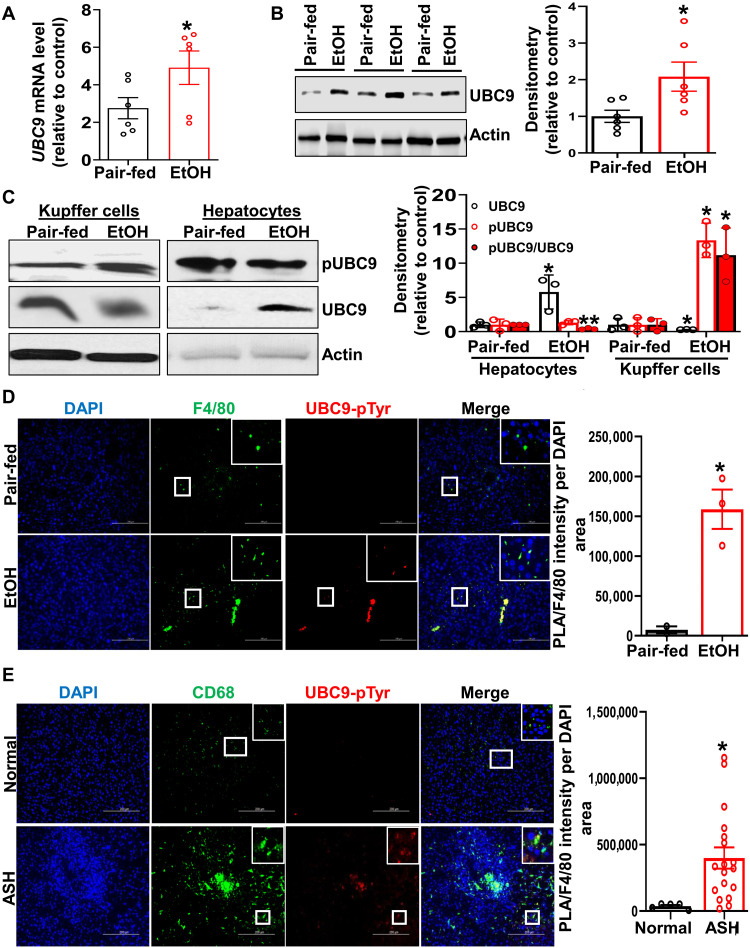
UBC9 phosphorylation is exclusively induced in KCs upon alcohol exposure. (**A**) UBC9 mRNA (*n* = 6 per group) and (**B**) protein levels from pair-fed and ethanol-fed mice livers (*n* = 6 per group). Means ± SE. *P < 0.03 (mRNA) and *P < 0.04 (proteins) versus pair-fed (two-tailed *t* test). (**C**) Western blot analysis of phosphorylated UBC9 (pUBC9) and total UBC9 expression in isolated KCs and hepatocytes from NIAAA livers or pair-fed control livers. Means ± SE (*n* = 3 per group). *P < 0.05 and **P < 0.001 versus pair-fed (two-tailed *t* test). (**D**) Representative immunofluorescence images showing UBC9 phosphorylation in liver sections from pair-fed and ethanol-fed mice. Staining includes F4/80 (KCs marker; green), UBC9 (red), and pTyr PLA probe (far-red). Nuclei were counterstained with 4′,6-diamidino-2-phenylindole (DAPI; blue). Scale bars, 200 μm. Results are presented as means ± SE (*n* = 3 per group). *P < 0.05 versus pair-fed (two-tailed *t* test). (**E**) Proximity ligation assay (PLA) demonstrating increased pUBC9 signal in CD68^+^ macrophages from human alcohol-associated steatohepatitis (ASH; *n* = 18) liver samples compared with normal livers (*n* = 5), Scale bars, 200 μm. Data are presented as means ± SE (*n* = 5 to 18). *P < 0.03 versus normal (two-tailed *t* test).

Chronic alcohol consumption is known to activate KCs (*20*), triggering inflammatory responses and metabolic dysregulation in hepatocytes. Previously, we demonstrated phosphorylation of UBC9 at serine-71 (S71) is induced in cancer cells (*12*). Therefore, to determine whether alcohol exposure alters UBC9 phosphorylation in liver-resident macrophages, we evaluated UBC9 expression in livers from the National Institute on Alcohol Abuse and Alcoholism (NIAAA) model. The expression level of UBC9 was significantly elevated in ethanol-fed mice compared with pair-fed controls (Fig. 1, A and B), confirming our previous results in 3-day ethanol-fed model (*21*). Notably, the level of phosphorylated UBC9 (pUBC9) exclusively increased in KCs upon ethanol treatment, but no change was observed in hepatocytes (Fig. 1C). However, we observed a decrease in pUBC9/UBC9 ratio in hepatocytes, suggesting a cell-specific role of UBC9 phosphorylation. Using phospho-peptide mapping analysis, we found that UBC9 is phosphorylated at two serine residues (S2 and S7) and one tyrosine residue (Y68) (Table 1) in LPS-activated KCs and KCs derived from alcohol-fed mice. The S2 and S7 residues in UBC9 were phosphorylated in both normal and activated KCs, whereas Y68 phosphorylation was specific to activated KCs (Table 1). UBC9 was phosphorylated at the S2 and S7 residues only in control but not alcohol-treated hepatocytes. Y68 phosphorylation was not detected in hepatocytes (Table 1). Tandem mass spectrometry (MS/MS) spectral analysis in KCs confirmed the loss of phosphate mass of 80 Da in the Y68 residue of the DDY*PSSPPK peptide (fig. S1, A and B). A second unrelated phospho-peptide (SGIALSRLAQER) was detected with a low ion score, indicating low confidence intensity (fig. S1, A and B). Using proximity ligation assay (PLA), we further examined the tyrosine phosphorylation of UBC9 (UBC9-p-Tyr) in conjunction with F4/80 (KC marker) in liver sections from pair-fed control and ethanol-fed mice. Confocal microscopy revealed a significant increase in UBC9-p-Tyr staining in F4/80^+^ KCs from ethanol-fed mice compared with controls (Fig. 1D). To validate the clinical relevance of these findings, we measured UBC9 phosphorylation in human alcohol-associated steatohepatitis (ASH) liver samples. PLA staining showed a significant increase in UBC9-p-Tyr intensity in CD68^+^ macrophages in ASH livers compared with pair-fed livers (Fig. 1E and fig. S2). This finding reinforces the observation that UBC9 phosphorylation is induced in hepatic macrophages in response to alcohol.

**Table 1.** Phospho-peptide mapping. Phospho-peptide mapping of UBC9 from control or LPS-treated KCs and KCs or hepatocytes from pair-fed or alcohol-fed (EtOH) mice is described in Materials and Methods. The observed mass is the mass of the peptide after a neutral loss of phosphate that reduces the mass by 98 Da due to S/T phosphorylation and by 80 Da due to Y phosphorylation.

Cell type/treatment		Observed mass	Start seq.	End seq.	Phospho-peptide sequence	Modification	Ion score
**KCs**	**Control**	1293.8412	66	76	DDYPSSPPKCK	Carbamidomethyl	9
		1300.6512	2	13	SGIALSRLAQER	Phospho (ST)[2,7]	12
		1314.7957	19	30	DHPFGFVAVPTK		10
		1360.6981	50	61	GTPWEGGLFKLR		10
	**LPS**	1373.8278	66	76	DDYPSSPPKCK	Carbamidomethyl (C)[10], Phospho (Y)[3]	4
		1085.6381	66	74	DDYPSSPPK	Phospho (Y)[3]	11
		1085.6592	66	74	DDYPSSPPK	Phospho (ST)[5]	4
		1293.785	66	76	DDYPSSPPKCK	Carbamidomethyl (C)[10]	8
		1380.7402	2	13	SGIALSRLAQER	Phospho (ST)[2,7]	0
	**Pair-fed**	903.4732	142	147	VEYEKR	Phospho (Y)[3]	5
		994.5279	1	8	MSGIALSR	Phospho (ST)[2,7]	12
		1527.9486	1	13	MSGIALSRLAQER	Oxidation (M)[1], Phospho (ST)[7]	9
	**EtOH**	994.5227	1	8	MSGIALSR	Phospho (ST)[2,7]	10
		1394.7563	19	30	DHPFGFVAVPTK	Phospho (ST)[11]	5
		1085.62	66	76	DDYPSSPPK	Phospho (Y)[3]	9
**Hepatocytes**	**Pair-fed**	994.5286	1	8	MSGIALSR	Phospho (ST)[2,7]	15
		1010.524	1	8	MSGIALSR	Oxidation (M)[1], Phospho (ST)[2,7]	8
		1085.6517	66	74	DDYPSSPPK	Phospho (Y)[3]	5
		1316.6804	66	76	DDYPSSPPKCK	Phospho (Y)[3]	9
		1380.6936	2	13	SGIALSRLAQER	Phospho (ST)[1]	10
	**EtOH**	930.5202	1	8	MSGIALSR	Oxidation (M)[1], Phospho (ST)[2]	10
		1373.7334	66	76	DDYPSSPPKCK	Phospho(ST)[5]	5
		1380.6818	2	13	SGIALSRLAQER	Phospho (ST)[1]	6
		2967.5647	83	101	PNVYPSGTVCLSILEED	Phospho(ST)[13]	3
		3141.6152	76	101	KFEPPLFHPNVYPSGTV	Phospho (ST)[17]	1

### UBC9 phosphorylation at Y68 residue causes inflammatory response in KCs

**Fig. 2.**
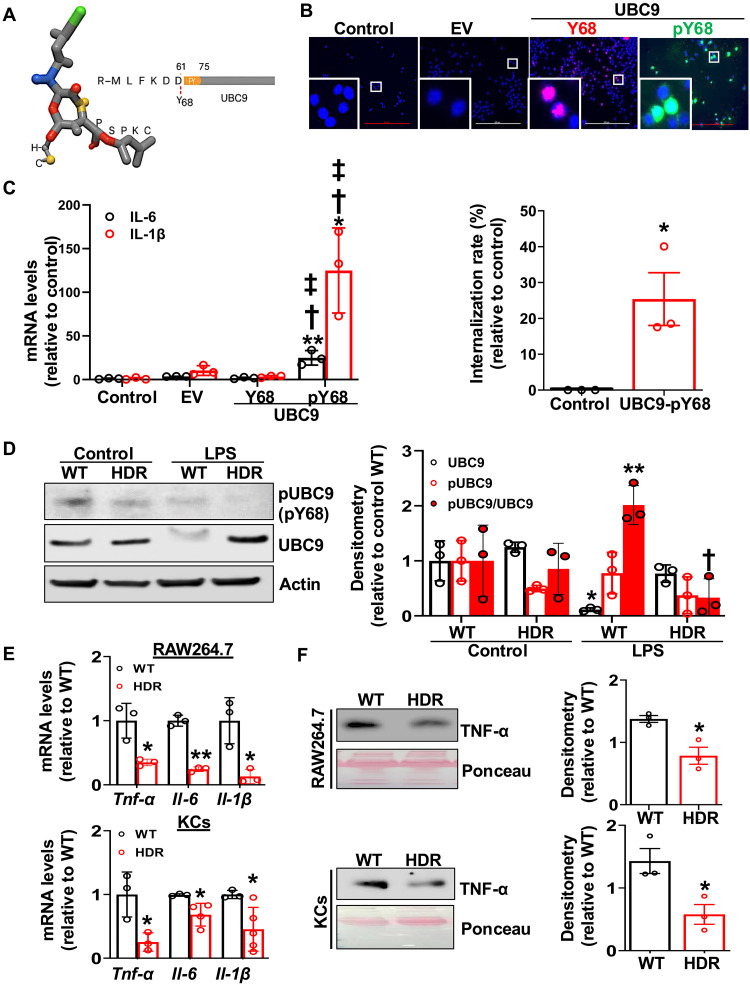
Phosphorylation of UBC9 at Y68 residue promotes NF-κB–mediated proinflammatory cytokine expression in macrophages. (**A**) Three-dimensional molecular model of a custom UBC9 fluorescein isothiocyanate (FITC)–labeled d-peptide phosphomimetic at tyrosine 68 (UBC9-pY68). Atoms are colored as follows: carbon (gray), oxygen (red), nitrogen (blue), phosphorus (yellow), and the N-terminal fluorescein (green). The phosphorylated d-tyrosine residue is shown prominently, representing the key posttranslational modification driving UBC9-dependent proinflammatory signaling. (**B** and **C**) RAW264.7 cells were transfected with 1 μg of empty vector (EV), 1 μg of UBC9-HA tagged vector (UBC9-Y68), and 5 μg of UBC9-pY68 peptide. (B) Y68 (red) and pY68 (green) cellular intake was visualized by immunofluorescence (20×) while nuclei were stained with DAPI (blue). Scale bars, 200 μm. Results are presented as means ± SE (*n* = 3 per group). *P < 0.03 versus control (two-tailed *t* test). (C) mRNA levels of *Il-6* and *Il-1*β were measured by RT-PCR. Means ± SE (*n* = 3 per group). **P < 0.001 versus control, *P < 0.01 versus control, †*P* < 0.01 versus EV, ‡*P* < 0.01 versus UBC9-Y68 [one-way analysis of variance (ANOVA)]. RAW264.7 cells and KCs were CRISPR-edited with UBC9 Y68 (HDR) or Scrambled [wild-type (WT)]. (**D**) Phosphorylated and total UBC9 were analyzed by Western blot, and actin was used as housekeeping. pUBC9 level was detected by phospho-specific UBC9 antibody as indicated in methods (*n* = 3 per group). *P < 0.03 and **P < 0.01 versus control WT, †*P* < 0.001 versus LPS WT (two-way ANOVA). (**E**) mRNA levels *Tnf-*α, *IL-6*, and *IL-1*β were measured by RT-PCR. Means ± SE (*n* = 3 per group). For RAW264.7 cells: *P < 0.02 and **P < 0.001 versus WT (two-tailed *t* test). For KCs: *P < 0.03 versus WT (two-tailed *t* test). (**F**) Quantification of secreted TNF-α from culture medium from RAW264.7 cells and KCs expressing WT or Y68F-mutant UBC9 by Western blot. Data are presented as means ± SE (*n* = 3 per group). *P < 0.03 versus WT (two-tailed *t* test).

To investigate whether UBC9 phosphorylation at tyrosine-68 leads to inflammatory activation of KCs, we used a phospho-mimetic peptide (UBC9-pY68) that was designed to structurally mimic the phosphorylation at Y68 residue (Fig. 2A) and was internalized into cells, as evidenced by fluorescein isothiocyanate (FITC) signal in the cytoplasm (Fig. 2B). Treatment of RAW264.7 cells with the peptide showed a strong induction of proinflammatory cytokine expression, including IL-6 and IL-1β, compared with control, empty vector, UBC9-Y68–treated cells (Fig. 2C). This finding indicates that Y68 phosphorylation can cause inflammatory gene activation. To examine the endogenous level of pUBC9 Y68, we generated and evaluated a custom antibody against phosphorylated Y68 residue (fig. S3, A and B). The use of this antibody showed a substantial increase of phosphorylation of UBC9 Y68 in RAW264.7 cells treated with LPS, as indicated by increased pUBC9/UBC9 ratio. At the same time, total UBC9 level decreased, as we previously reported (fig. S3C) (*10*). Impressively, UBC9 CRISPR gene–edited RAW264.7 cells carrying a nonphosphorylatable phenylalanine-68 (F68) (fig. S4) showed a lowered pUBC9/UBC9 ratio (Fig. 2D). This result confirms the specificity of the pUBC9Y68 antibody and the reduction of phosphorylation at this site. Consistent with this, gene editing resulted in markedly decreased gene expression of proinflammatory markers although cells were treated with LPS compared with wild-type (WT) cells (Fig. 2E). In addition, we found that the Y68F mutation significantly reduced LPS-induced secretion of TNF-α, IL-6 and IL-1β from RAW264.7 cells and KCs (Fig. 2F). Together, these findings demonstrate that phosphorylated Y68 residue is critical on UBC9 that may lead to inflammatory response.

### Phosphorylation of UBC9 at Y68 is mediated by SRC kinase

**Fig. 3.**
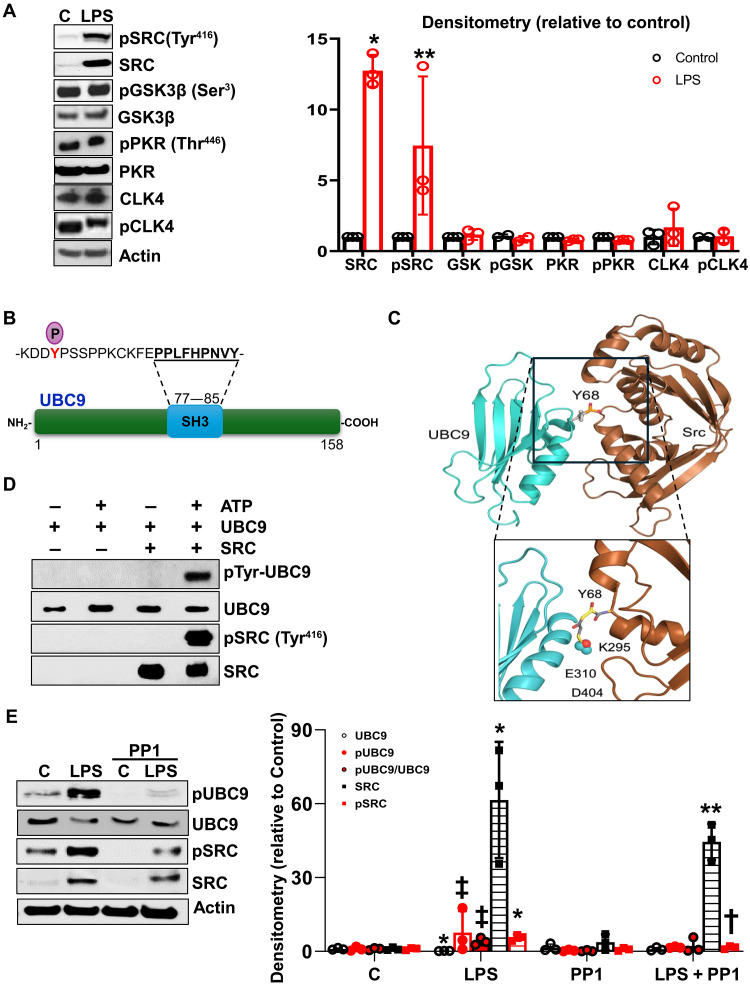
SRC kinase mediates phosphorylation of UBC9 at Y68 in macrophages. RAW264.7 cells were treated with SRC inhibitor (PP1, 20 μM, 2 hours) followed by LPS treatment (500 ng/ml) for 16 hours. (**A**) The levels of UBC9 interacting kinases (SRC, pSRC Tyr^416^, GSK3β, pGSK3β Ser^3^, PKR, pPKR Thr^446^, CLK4, and pCLK4) and actin, as housekeeping, were analyzed by Western blot. Means ± SE. *P < 0.003 versus control SRC (*n* = 5 per group, two-tailed *t* test); **P < 0.04 versus control pSRC (*n* = 3 per group, two-tailed *t* test). (**B**) Schematic representation of SRC binding motif (SH3: 77 to 85 amino acids) in UBC9 protein sequence. (**C**) A three-dimensionally rendered structural model illustrates the potential interface between UBC9 (turquoise) and SRC kinase (brown), emphasizing the putative interaction site at tyrosine-68 (Y68). The phosphorylated Y68 residue (pY68) is shown as a ball-and-stick representation, with the phosphate group highlighted in red and yellow. The visualization depicts secondary structure elements, including α helices and β sheets, for both proteins. (**D**) In vitro kinase assay to test the efficacy of the active form of pSRC (Tyr^416^) to phosphorylate the recombinant UBC9 at tyrosine residues (*n* = 3 per group). (**E**) The effect of SRC inhibition was analyzed by Western blot, incubating the membrane with antibodies against phosphorylated pSRC (Tyr^416^), total SRC, pUBC9Y68 (E25961), and total UBC9. Means ± SE (*n* = 3 per group). UBC9: *P < 0.02 versus C (one-way ANOVA); pUBC9: ‡*P* < 0.02 versus PP1 (one-way ANOVA); pUBC9/UBC9: ‡*P* < 0.02 versus PP1 (one-way ANOVA); SRC: *P < 0.02 versus C, **P < 0.05 versus C (one-way ANOVA); pSRC: *P < 0.02 versus C, †*P* < 0.02 versus LPS (one-way ANOVA).

By UBC9 antibody pulldown followed by mass spectrometry (MS) and analysis of key binding partners, we identified several kinases to differentially interact with UBC9 in livers from ethanol-fed versus pair-fed mice. The label-free quantification (LFQ) intensity of unique peptides corresponding to specific kinases was compared between pair-fed and ethanol-fed liver samples. Among the interacting kinases, SRC exhibited a 5.1-fold increased interaction in ethanol livers compared with pair-fed controls (Table 2). Increased activity of SRC (pSRC-Y416 phosphorylation) was observed in LPS-treated RAW264.7 cells (Fig. 3A). UBC9 contains a PXXP motif [Src homology 3 (SH3) recognition motif], which can interact with the SH3 domain of SRC (Fig. 3B) (*22*, *23*). Using ClusPro to create the docking model and Pymol software, we made a visual mock-up to simulate the spatial proximity of UBC9 Y68 residue to the SRC kinase domain (Fig. 3C). In this model, which was verified using GTP 4.0 AI, Y68 residue is positioned near the catalytic cleft of the SRC kinase domain [~6.2 Å from the SRC active site (K295/E310/D404)]. This spatial orientation is essential for phosphorylation: SRC must physically access the hydroxyl group of tyrosine to catalyze the transfer of a phosphate group from adenosine 5′-triphosphate (ATP) (*24*). This motif appears aligned with the SH3 domain of SRC, stabilizing the interaction and potentially anchoring UBC9 in an orientation that favors Y68 access. SH3-mediated docking increases substrate specificity by localizing the substrate to the kinase. SRC kinases preferentially phosphorylate tyrosine residues within specific sequence motifs. UBC9 surrounding Y68 fits a plausible substrate motif. Y68 lies in a solvent-accessible, flexible loop, which is ideal for phosphorylation because buried or rigid residues are not typically modified. This hypothesized interaction was then confirmed using the in vitro kinase phosphorylation assay (Fig. 3D). To determine whether SRC mediates UBC9 phosphorylation at Y68, we used pharmacological inhibition approach. Treatment of RAW264.7 with the SRC inhibitor PP1 prevented LPS-induced UBC9 phosphorylation (Fig. 3E). These findings establish SRC kinase as the primary regulator of UBC9 phosphorylation at Y68 in macrophages.

**Table 2.** UBC9 interacting kinases in ethanol-fed and pair-fed mouse livers. Kinases interacting with UBC9 were pulled down using UBC9 antibody followed by MS, as described in Materials and Methods. Label-free quantification (LFQ) of these kinases was compared between pair-fed and ethanol-fed groups. The data represents the LFQ fold change of ethanol-fed group compared with that of pair-fed control group.

Protein names	LFQ intensity fold change (ETOH-fed versus pair-fed)
Neuronal proto-oncogene tyrosine-protein kinase Src	5.1
Glycogen synthase kinase-3 beta (GSK3β)	2.9
Serine/threonine-protein kinase 3 and 4 (SKT3 and SKT4)	1.2
Tyrosine-protein kinase BTK	0.5
Tyrosine-protein kinase Fes/Fps	0.8
Dual specificity protein kinase CLK4; dual-specificity protein kinase CLK1	4.7
Interferon-induced, double-stranded RNA-activated protein kinase (Eif2ak2)	3.6
Serine/threonine-protein kinase PAK 1	0.32
Tyrosine-protein kinase SYK	1.1
Tyrosine-protein kinase BTK	0.5

### SRC interacts with UBC9 in human and mouse macrophages in response to inflammatory and alcohol-related stimuli

**Fig. 4.**
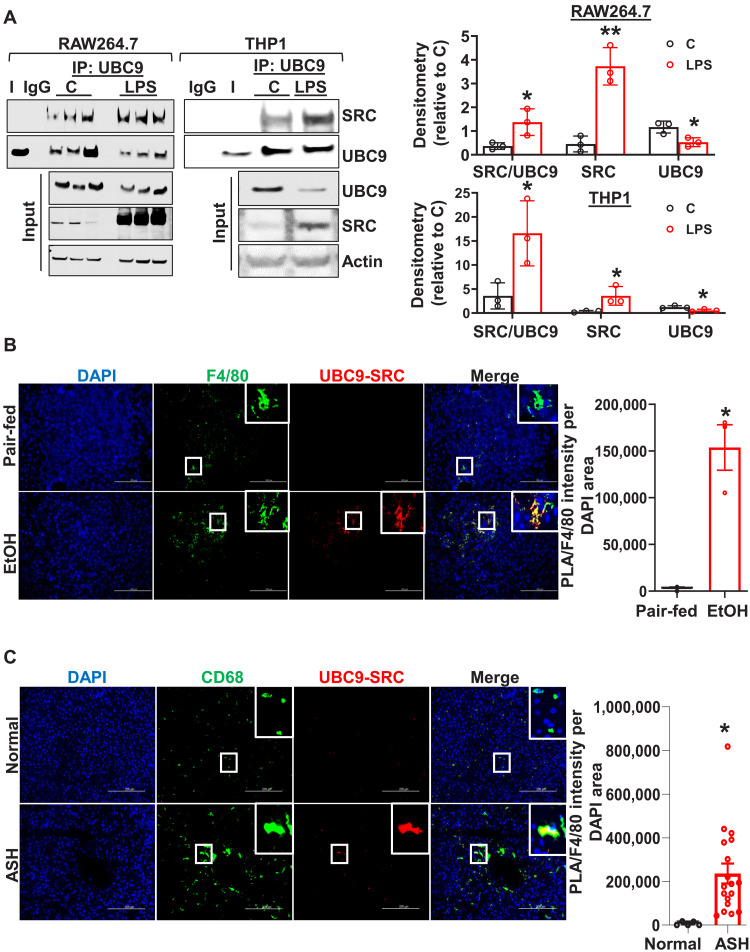
UBC9 interacts with SRC in murine and human ALD. (**A**) UBC9 was immunoprecipitated with SRC in RAW264.7 and THP1 macrophages when treated with LPS (500 ng/ml) for 16 hours. Right panels show densitometric analysis of UBC9/SRC interaction, total UBC9, and UBC9 normalized versus control (C). Data are presented as means ± SE. For RAW264.7 (*n* = 3 per group): *P < 0.05 and **P < 0.005 versus control (two-tailed *t* test). For THP1 (*n* = 3 per group): *P < 0.05 versus control (two-tailed *t* test). (**B**) PLA images showing the physical interaction between UBC9 and SRC (red) in F4/80^+^ KCs (green) in liver sections from the NIAAA model. DAPI was used as a counterstain (blue). Scale bars, 200 μm. PLA-positive cells were quantified by ImageJ. Means ± SE (*n* = 3 per group). *P < 0.02 versus pair-fed (two-tailed *t* test). (**C**) PLA detection of UBC9 interacting with SRC (red) in CD68^+^ KCs (green) in liver samples from control (healthy; *n* = 5) versus alcoholic steatohepatitis livers (ASH; *n* = 18). Scale bars, 200 μm. Means ± SE (*n* = 5 to 18). *P < 0.0001 versus normal (two-tailed *t* test).

LPS has been reported to induce SRC expression and promote TNF-α secretion in macrophages (*25*). To investigate whether inflammatory stimuli promote the interaction between SRC and UBC9 in macrophages and KCs, we examined their interaction in vitro and vivo. We found that LPS significantly induced SRC and UBC9 immunoprecipitation in both RAW264.7 and human THP1 monocytes compared with untreated controls (Fig. 4A). This induction could be due to the abundance of SRC and increased interaction with UBC9, as our interactome data show. This finding was further confirmed by PLA assay, which revealed a strong SRC and UBC9 interaction in F4/80^+^ KCs from ethanol-fed mice (Fig. 4B) and CD68^+^ KCs from human ASH livers compared with relative controls (Fig. 4C and fig. S5). These findings support a role for SRC and UBC9 complex signaling in inflammatory activation of liver-resident macrophages.

### UBC9 phosphorylation exacerbates alcohol-induced liver injury

**Fig. 5.**
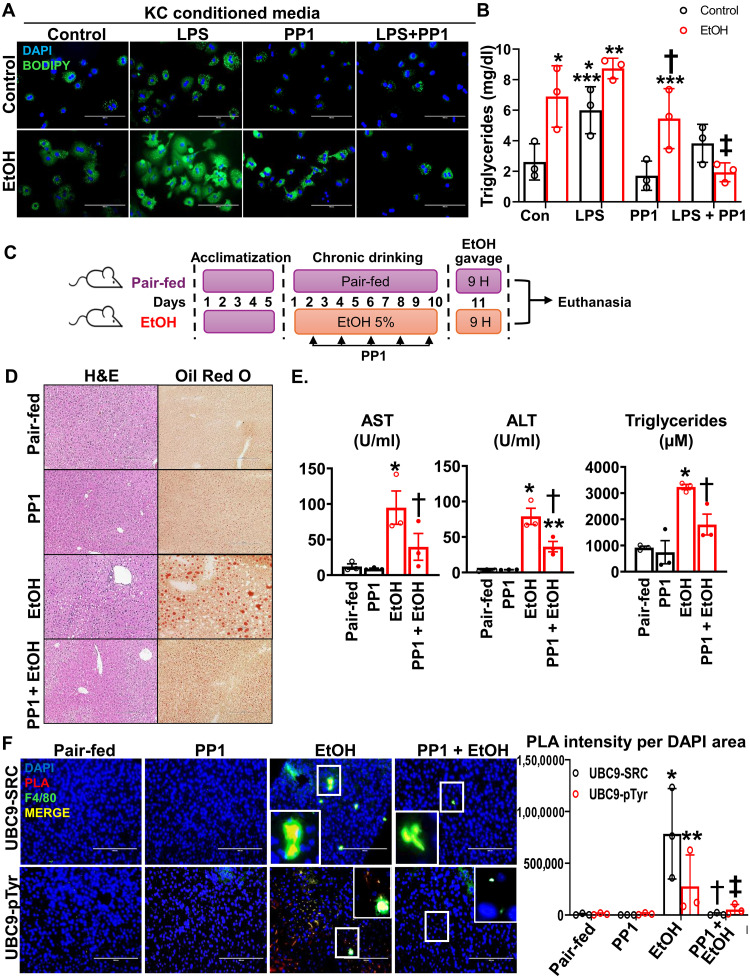
UBC9 phosphorylation in KCs enhances hepatic lipogenesis and triglyceride accumulation. Mouse hepatocytes were cultured with conditioned medium from LPS (500 ng/ml per 16 hours)– and/or PP1 (20 μM, 2 hours)–treated KCs and cotreated with ethanol (100 mM) for 24 hours. (**A**) Lipid accumulations were visualized by BODIPY staining (*n* = 3 per group). Scale bars, 200 μm. (**B**) Triglycerides measurement in hepatocytes cultured with conditioned medium from LPS/PP1-treated KCs. Data are presented as means ± SE (*n* = 3 per group). *P < 0.01 versus control, **P < 0.05 versus control LPS, ***P < 0.01 versus control PP1, †*P* < 0.01 versus ethanol (EtOH) LPS, ‡*P* < 0.0001 versus EtOH LPS (two-way ANOVA). (**C**) Schematic of in vivo PP1 treatment experimental design. H, hours. (**D**) Representative hematoxylin and eosin (H&E) and Oil Red O–stained liver sections from control, ethanol-fed, and PP1-treated mice. Scale bars, 200 μm (*n* = 3 per group). (**E**) Plasma ALT and AST levels and liver triglyceride levels in ethanol-fed mice with or without PP1 treatment (1.5 mg/kg, every other day for 10 days). Means ± SE (*n* = 3 per group). *P < 0.01 versus pair-fed, †*P* < 0.05 versus EtOH (AST; one-way ANOVA); *P < 0.0001 versus pair-fed, **P < 0.01 versus pair-fed, †*P* < 0.01 versus EtOH (ALT; one-way ANOVA); *P < 0.001 versus pair-fed, †*P* < 0.02 versus EtOH (triglycerides; one-way ANOVA). (**F**) PLA images showing the physical interaction between UBC9 and SRC (red) in F4/80^+^ KCs (green) in liver sections from PP1-treated NIAAA model. DAPI was used as a counterstain (blue). Scale bars, 200 μm. PLA intensity per DAPI area was quantified by ImageJ. Means ± SE (*n* = 3 per group). UBC9-SRC: *P < 0.01 versus pair-fed, †*P* < 0.01 versus EtOH (one-way ANOVA); UBC9-pTyr: **P < 0.02 versus pair-fed, ‡*P* < 0.05 versus EtOH (one-way ANOVA).

Because NF-κB signaling has been implicated in lipid metabolism and SRC induced NF-κB activation through tyrosine phosphorylation of IκBα and p65 NF-κB in RAW 264.7 cells (*26*, *27*), we next investigated whether UBC9 phosphorylation contributes to alcohol-induced liver damage. Conditioned medium from KCs cultured after isolation from LPS or ethanol-fed mice induced lipid accumulation in hepatocytes, as shown by increased BODIPY staining. This effect was blunted when these LPS or alcohol-exposed KCs were cotreated with PP1 (SRC inhibitor), suggesting that SRC activity mediates lipid signaling in hepatocytes (Fig. 5A), as was confirmed by the triglyceride levels (Fig. 5B). Histological examination of PP1-treated ethanol-fed mice liver (Fig. 5C) confirmed that pharmacological inhibition of SRC prevented alcohol-induced hepatic steatosis and injury (Fig. 5D). Further, plasma alanine aminotransferase (ALT) and aspartate aminotransferase (AST) levels, as well as hepatic triglycerides, were significantly increased by ethanol and significantly ameliorated by PP1 (Fig. 5E). In addition, PLA assay confirmed that ethanol induced SRC/UBC9 complex formation as well as UBC9 tyrosine phosphorylation in F4/80^+^ KCs, while PP1 cotreatment markedly reduced it (Fig. 5F). These findings suggest that SRC activity mediates UBC9 phosphorylation and its interaction with SRC in response to ethanol treatment.

### Targeting UBC9 phosphorylation as a therapeutic strategy for ALD

**Fig. 6.**
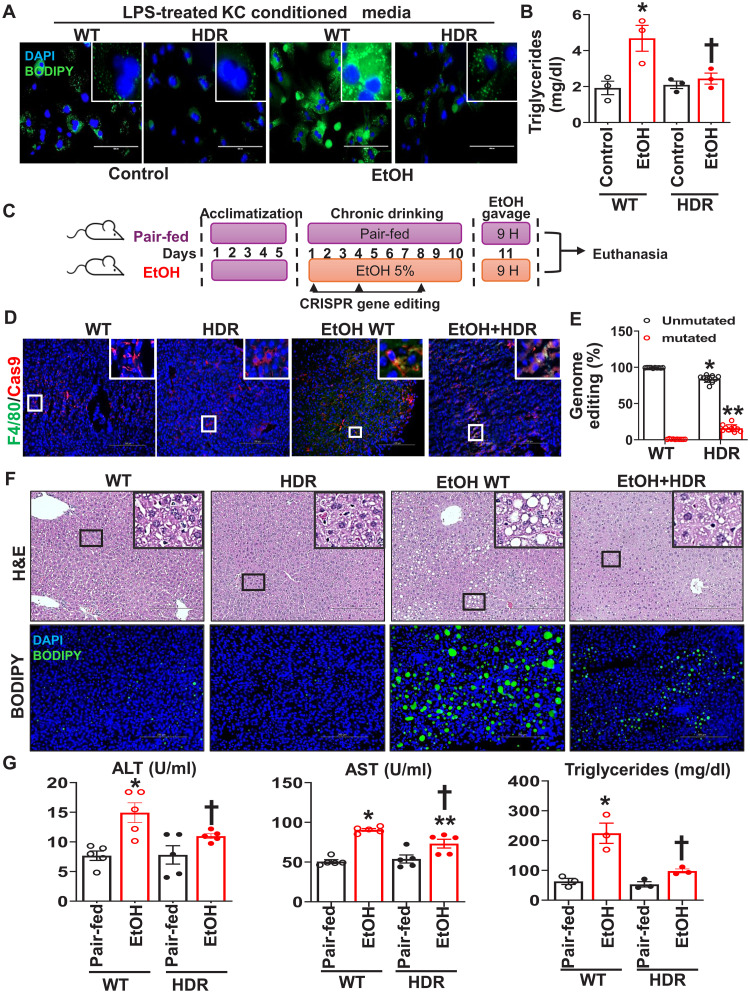
CRISPR-mediated Y68F mutation of UBC9 protects against ethanol-induced steatosis. Mouse hepatocytes were cultured with conditioned medium from CRISPR-edited, LPS-treated KCs (500 ng/ml per 16 hours) and were further treated with ethanol (100 mM) for 24 hours. (**A**) Lipid droplets were stained with BODIPY (scale bars, 200 μm) and (**B**) quantified by triglycerides assay. Data are presented as means ± SE (*n* = 3 per group). *P < 0.01 versus control WT, †*P* < 0.01 versus EtOH WT (two-way ANOVA). (**C**) Schematic of in vivo CRISPR-Cas9 editing experimental design. H, hours. (**D**) Immunofluorescence of Cas9 and F4/80 to verify the transduction efficiency in KCs from liver sections of NIAAA mice (*n* = 6 per group). Scale bars, 200 μm. (**E**) Next-generation sequencing to determine CRISPR efficiency as in methods. Data are presented as means ± SE (*n* = 10 per group). *P < 0.0001 versus WT unmutated, **P < 0.0001 versus WT mutated (two-way ANOVA). (**F**) Representative H&E and BODIPY staining in livers of ethanol-fed WT and Y68F-mutant mice. Scale bars, 200 μm (*n* = 6 per group). (**G**) Plasma ALT (*n* = 5 per group), AST (*n* = 5 per group), and hepatic triglyceride (*n* = 3 per group) levels in CRISPR-edited and WT mice. Means ± SE. *P < 0.001 versus pair-fed WT, †*P* < 0.05 versus EtOH WT (ALT; two-way ANOVA); *P < 0.0001 versus pair-fed WT, **P < 0.01 versus pair-fed WT, †*P* < 0.01 versus EtOH WT (AST; two-way ANOVA); *P < 0.001 versus pair-fed WT, †*P* < 0.01 versus EtOH WT (triglycerides; two-way ANOVA).

To assess the functional role of pUBC9 in ethanol-induced liver injury, we used CRISPR-Cas9 technology to mutate Y68 to F68 [homology-directed repair (HDR)], which prevented UBC9 phosphorylation in in vitro and in vivo KCs. BODIPY staining was significantly increased in hepatocytes exposed to conditioned medium from ethanol-treated WT KCs but was reduced when medium derived from HDR-edited KCs (Fig. 6A). Additionally, triglyceride measurements confirmed that ethanol-induced lipid accumulation was decreased in hepatocytes when exposed to conditioned medium from HDR-edited KCs compared with WT (Fig. 6B). CRISPR genome editing targeting UBC9 phospho-site in liver-resident macrophages (Fig. 6C) was performed by a Cas9 enzyme under the control of the CD68 promoter. Macrophage-specific CRISPR was confirmed by costaining of Cas9 with F4/80 in CRISPR-edited liver sections (Fig. 6D). The efficiency of HDR in mice was assessed by next-generation amplicon sequencing (NGS), as described in methods (Fig. 6E). Suppressing the UBC9 phosphorylation in KCs effectively protected mice from ethanol-induced liver damage, as evidenced by reduced ALT and AST levels and improved liver histology (Fig. 6, F and G). These findings described UBC9 phosphorylation as a critical mediator of alcohol-induced liver injury.

### Alcohol-induced UBC9 phosphorylation promotes lipogenic gene expression via NF-κB activation in vivo

**Fig. 7.**
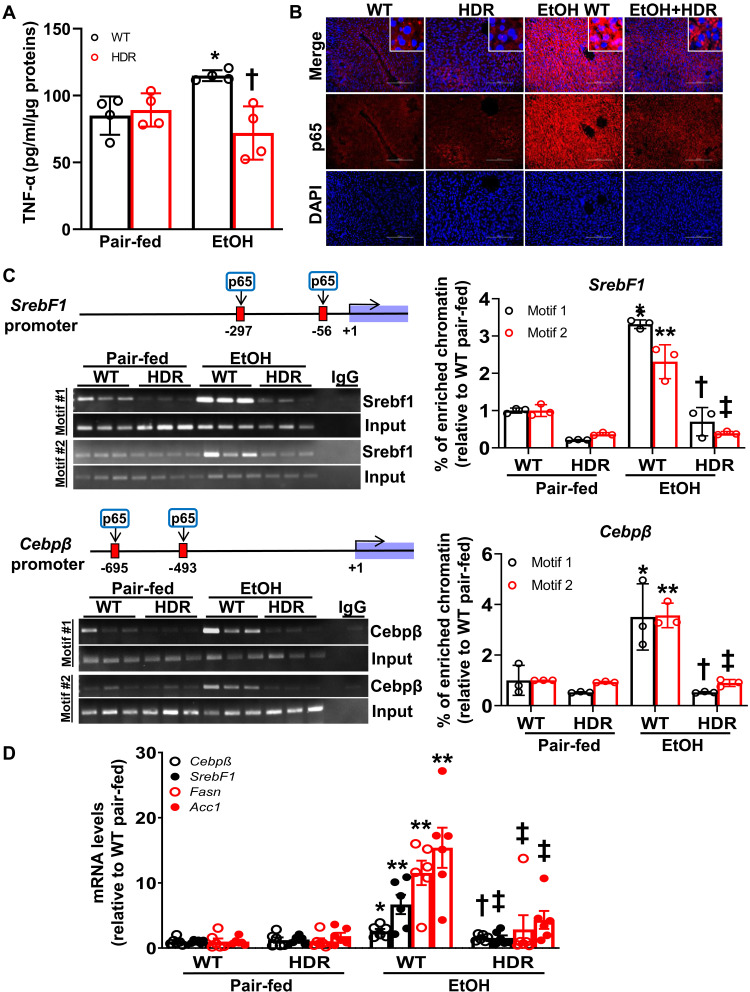
pUBC9 promotes de novo lipogenesis in vivo. Mice were ethanol-fed and treated with lentivirus to gene edit UBC9 Y68 residue to F68 (HDR) in KCs compared with WT as described in Materials and Methods. (**A**) Plasmatic level of TNF-α was measured by enzyme-linked immunosorbent assay (*n* = 4 per group). *P < 0.02 versus WT control, †*P* < 0.001 versus WT EtOH (two-way ANOVA). (**B**) Immunofluorescence of p65 (red) in liver sections (20×). Nuclei were counterstained with DAPI (blue). Scale bars, 200 μm (*n* = 6). (**C**) ChIP analysis of p65 binding to *SrebF1* (motif 1: −56/−45; motif 2: −297/−286) and *Cebp*β promoters (motif 1: −493/−484; motif 2: −695/−686) from WT and HDR mouse livers with or without ethanol exposure. Schematic diagrams of p65 binding motifs. Right panels show quantification of p65 enrichment relative to pair-fed WT mice. Data are presented as means ± SE (*n* = 3 per group). *SrebF1* promoter: *P < 0.0001 (motif 1) and **P < 0.01 (motif 2) versus pair-fed WT; †*P* < 0.0001 (motif 1) and ‡*P* < 0.0001 (motif 2) versus EtOH WT (two-way ANOVA); *Cebp*β promoter: *P < 0.01 (motif 1) and **P < 0.0001 (motif 2) versus pair-fed WT; †*P* < 0.01 (motif 1) and ‡*P* < 0.0001 (motif 2) versus EtOH WT (two-way ANOVA). (**D**) mRNA levels of *Cebp*β, *SrebF1, Fasn*, and *Acc1* were measured by RT-PCR from mouse livers. Means ± SE (*n* = 6 per group). *Cebp*β mRNA: *P < 0.01 versus pair-fed WT, †*P* < 0.05 versus EtOH WT (two-way ANOVA); *SrebF1* mRNA: **P < 0.0001 versus pair-fed WT, ‡*P* < 0.01 versus EtOH WT (two-way ANOVA); *Fasn* mRNA: **P < 0.0001 versus pair-fed WT, ‡*P* < 0.01 versus EtOH WT (two-way ANOVA); *Acc1* mRNA: **P < 0.0001 versus pair-fed WT, ‡*P* < 0.01 versus EtOH WT (two-way ANOVA).

Because UBC9 phosphorylation induces NF-κB transactivation activity in KCs (*10*), we examined whether this pathway plays a role in hepatic lipogenesis in vivo. We found induced levels of TNF-α in WT ethanol-fed mice, but not in UBC9 Y68F gene-edited mice (HDR) (Fig. 7A), suggesting a negative impact on inflammatory signaling. Furthermore, p65 immunofluorescence staining of liver sections exhibited a strong nuclear localization in WT upon ethanol treatment, which was inhibited in HDR mice (Fig. 7B). To confirm that pUBC9 leads to NF-κB transactivation, we performed chromatin immunoprecipitation (ChIP) assays to check the binding of p65 to its known promoter targets, *Srebf1* and *Cebp*β (*28*, *29*). Ethanol treatment significantly increased p65 binding to *Srebf1* and *Cebp*β promoters compared with WT livers at the binding motifs near the transcriptional starting site (fig. S6, A and B). In contrast, p65 binding affinity was lowered in HDR mice (Fig. 7C). This decrease in p65 activity by HDR was also associated with reduced expression of alcohol-induced lipogenic genes, including *Srebf1*, *Cebp*β, *Fasn*, and *Acc1* upon UBC9-Y68F (Fig. 7D). Together, these findings proved that pUBC9 in KCs promotes NF-κB recruitment to lipogenic gene promoters, sustaining de novo lipogenesis in ALD.

## DISCUSSION

**Fig. 8.**
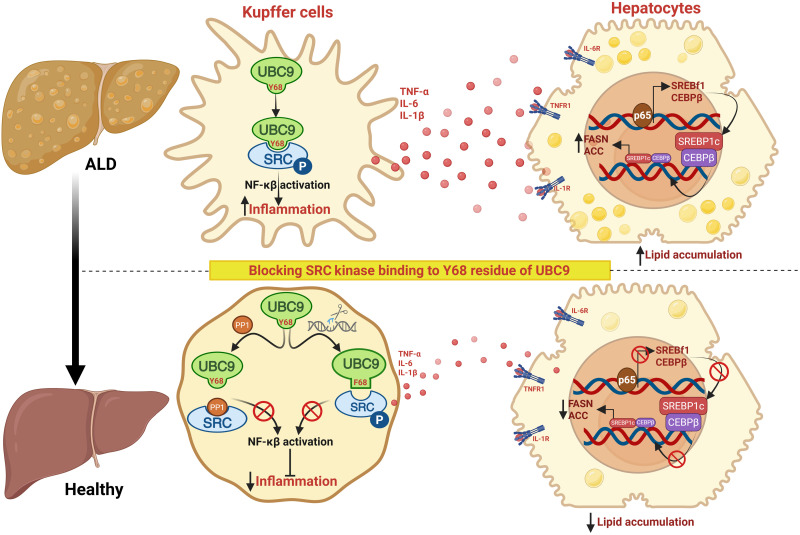
Proposed mechanism showing blocking SRC kinase binding to Y68 residue of UBC9 protects against ALD. In response to chronic alcohol exposure, gut-derived LPS activates KCs, leading to SRC kinase mediated phosphorylation of UBC9 at Y68 residue. This phosphorylation amplifies NF-κB signaling, leading to increased production of proinflammatory cytokines (TNF-α, IL-6, and IL-1β). These cytokines subsequently up-regulate the lipogenic transcription factors SREBP1c and CEBPβ, thereby inducing FASN and ACC that drive lipid accumulation in hepatocytes. In contrast, genetic or pharmacologic blockade of SRC kinase-mediated UBC9-Y68 phosphorylation (e.g., Y68F mutation or SRC activity inhibition) diminishes NF-κB signaling and cytokine production in KCs, thereby protecting against steatosis via suppressing lipogenic gene expression. Created in BioRender. Chandla, S. (2026) https://BioRender.com/lizzngv.

In this study, we identify SRC-mediated phosphorylation of UBC9 at Y68 residue as a critical regulatory event in ALD. Specifically, we demonstrate that pUBC9 is induced in KCs upon ethanol exposure, which generates NF-κB–dependent inflammatory cytokine production and hepatic lipogenesis. Our findings establish SRC-mediated UBC9 phosphorylation as a mechanistic link between KC activation and hepatocellular injury in ALD. Additionally, we demonstrate that blocking SRC and UBC9 binding, through either pharmacological inhibition of SRC or CRISPR-mediated mutation of Y68, prevents ethanol-induced liver inflammation, steatosis, and injury, highlighting the therapeutic potential of this pathway in ALD (Fig. 8).

Our study shows that UBC9 phosphorylation at Y68 enhances NF-κB signaling in KCs, leading to higher expression of proinflammatory cytokines (TNF-α, IL-6, and IL-1β). NF-κB activation is a well-known driver of hepatic inflammation in ALD, and previous studies have implicated SUMOylation in modulating NF-κB activity (*30*–*32*). However, our data provide the first evidence that Y68 phosphorylation of UBC9, rather than SUMOylation alone, plays a key role in NF-κB activation in alcohol-exposed KCs.

A key finding of our study is revealed by kinase interactome analysis, which identified SRC as the main kinase responsible for UBC9 phosphorylation at Y68. This is supported by increased pSRC-Y416 levels in ethanol-fed mouse livers. SRC activation is a hallmark of alcohol-induced liver injury (*33*), and previous studies have linked SRC signaling to KC activation, oxidative stress, and hepatocyte apoptosis (*18*, *34*). Our results extend these findings by demonstrating that SRC directly phosphorylates UBC9, thereby promoting NF-κB activation and inflammatory cytokine production. Pharmacological inhibition of SRC by PP1 effectively reduces UBC9 phosphorylation, NF-κB activation, and inflammatory cytokine levels in ethanol-fed mice, confirming the role of SRC as an upstream regulator of UBC9 phosphorylation in ALD. Notably, SRC inhibition also prevents hepatic steatosis and triglyceride accumulation, suggesting that the UBC9-SRC axis is involved not only in inflammation but also in metabolic dysregulation.

Another important finding of our study is that SRC-induced UBC9 Y68 phosphorylation in KCs promotes hepatic lipogenesis through the activation of SREBP1c and CEBPβ (*35*, *36*). These transcription factors are key regulators of de novo lipogenesis, and their activation has been linked to the development of ALD (*37*, *38*). We show that CRISPR-Cas9–mediated Y68F mutation of UBC9 protects against alcohol-induced liver injury by lowering plasma ALT and AST levels, inflammatory cytokine production, and lipid accumulation. This indicates that SRC-mediated UBC9 Y68 phosphorylation is essential for KC-driven metabolic reprogramming in hepatocytes. Additionally, our ChIP analysis further demonstrates that SRC-mediated UBC9 phosphorylation enhances NF-κB recruitment to the SREBP1c and CEBPβ promoters, connecting inflammatory signaling with lipid metabolism. Because proinflammatory cytokines promote hepatic steatosis in ALD (*9*, *39*), our findings suggest that SRC-UBC9 binding acts as a molecular switch linking inflammation with lipid buildup in the liver. The liability of this study is that ethanol induces multiple inflammatory and metabolic pathways, and the SRC-UBC9 axis likely operates alongside other modulators of ALD progression. Future studies exploring these interactions will further establish the signaling framework and translational relevance of targeting this pathway.

Together, our findings suggest an emergent SRC-UBC9 axis that couples KC inflammatory activation to hepatic lipid accumulation in ALD. This mechanism potentially broadens the range of therapeutic targets and highlights the SRC-UBC9 axis as a prospective target in ALD.

## MATERIALS AND METHODS

### Study design

The goals of this study were twofold: to identify the upstream regulators of ALD progression in KCs in vivo and to validate the hypothesis that targeting SRC kinase or Y68 residue of UBC9 can reduce ethanol-mediated liver steatosis in vitro, in vivo, and in human ASH livers. We first confirmed ethanol-induced phosphorylation of UBC9 in KCs from NIAAA-model mice and then used phospho-peptide mapping to predict the phosphorylation site in KCs from NIAAA mice and LPS-treated mouse livers. This prediction was validated through phosphomimetic studies and CRISPR-Cas9 editing in RAW264.7 cells and primary KCs. To identify the kinase responsible, we conducted MS-based interactome analysis in ethanol-fed mouse livers and confirmed SRC as the key kinase using Western blotting, in vitro kinase assays, PP1 treatment in RAW264.7 cells, immunoprecipitation in RAW264.7 and THP-1 cells, and PLA assays in NIAAA mouse livers and human ASH livers. For in vivo studies, we used the NIAAA model because it is a well-established and clinically relevant model of ALD. The animal study was divided into two phases: (i) PP1 treatment and (ii) CRISPR gene editing of UBC9 Y68 in KCs. We then assessed downstream targets of the SRC-UBC9 pathway to determine whether these interventions alleviated ethanol-induced steatosis. Animals were randomly assigned to groups, and the study team was aware of the group allocation while performing the experiment. All in vitro and in vivo experiments were performed in 3 to 15 technical replicates, with biologically independent sample numbers indicated in each figure.

### Mice

#### NIAAA model

Procedures for the care and use of mice in this study were approved by the Institutional Animal Care and Use Committee at Cedars-Sinai Medical Center under an approved animal protocol [protocol no. 7952; principal investigator (PI): Maria Lauda Tomasi]. Three-month-old female C57BL/6 mice (Harlan, Indianapolis, IN, USA) were treated with an ethanol or pair-fed control diet following an established protocol for alcohol feeding (*40*) and housed in a temperature-controlled animal facility with 12-hour light-dark cycles. Briefly, mice were fed ad libitum with the Lieber-DeCarli liquid control diet (Bio-Serv, F1259) for 5 days and then randomly divided into two groups: the ethanol group (*n* = 4) was fed a liquid diet containing 5% ethanol (Bio-Serv, F1258) for 10 days, while the control group (*n* = 4) was pair-fed a control diet for 10 days. On day 11, mice in the ethanol group received single-binge ethanol feeding (5 g/kg, 20% ethanol), while mice in the control group received dextrin maltose. The gavage was performed early in the morning, and, after gavage, mice were kept on a control or ethanol diet and in cages with water. Nine hours after the gavage, mice were anesthetized and euthanized; blood was collected; and liver tissues were removed, snap frozen in liquid nitrogen, and stored at −80°C until analysis.

#### In vivo PP1 treatment

For PP1 injections, pair-fed or alcohol-fed mice as above were injected intraperitoneally with control RPMI medium or with PP1 (Sigma-Aldrich, 539571; 1.5 mg/kg body weight in RPMI) every alternate day till the end of the feeding period. After the final gavage of alcohol, mice were euthanized and processed for blood and tissue collection.

### Cell culture and treatments

#### Primary murine cells

Primary mouse hepatocytes (PMHs) and KCs were isolated from 10- to 12-week-old male and female C57BL/6 mice using collagenase perfusion, as previously described (*10*). Cells were cultured in Dulbecco’s modified Eagle’s medium (DMEM), supplemented with 2 mM glutamine, 10% fetal bovine serum (FBS), streptomycin sulfate (50 mg/ml), and 50 mM penicillin. After allowing the cells to adhere, the culture medium was replaced: after 2 hours for PMH and after 10 min for KCs. The cells were then maintained in the same medium for further experimentation.

#### Cell lines

RAW264.7 (mouse macrophage) was purchased from American Type Cell Collection (Manassas, VA) and cultured in DMEM supplemented with 10% FBS and antibiotics [2 mM glutamine, 50 mM penicillin, and streptomycin sulfate (50 mg/ml)].

#### PP1 and LPS treatment

Primary KCs and RAW264.7 cells (0.3 × 10^6^ cells per well) were seeded in six-well plate and treated with 20 μM PP1 (Sigma-Aldrich, 539571, lot no. 3150033) for 2 hours at 37°C with 5% CO_2_. Afterward, medium was renewed, and cells were incubated with LPS (500 ng/ml; Sigma-Aldrich, L3137-10MG) at 37°C with 5% CO_2_ overnight. The next day, the culture medium was saved for coculture experiments at −80°C, and the cells were used for biochemistry analysis.

### Coculture experiment

For coculture experiments, primary hepatocytes (0.4 × 10^6^ cells per well) were seeded in six-well plates. After adhesion, medium was replaced with KCs or RAW264.7 cell-conditioned medium collected either from the PP1 experiment or the CRISPR gene editing experiment. Next, hepatocytes were treated with 100 mM ethanol for 24 hours, and, then, cells were collected and saved at −80°C for further analysis.

### Construction of lentivirus for CRISPR gene editing

CRISPR-Cas9–mediated gene editing at the UBC9 gene locus to mutate the Y68 to F68 residue was performed using HDR. One guide RNA sequence (gRNA) recognized by SaCas9 was designed and cloned into pLV[Exp]-U6 > mUbe2i[gRNA#51] > CD68(short) > hCas9(ns):T2A:Puro by Vector Builder (Chicago, IL, USA), containing the SaCas9 gene under control of the KC-specific CD68 promoter. This plasmid was designated as gRNA/Cas9. A donor RNA to mutate the Y68 residue to F68 was also designed and cloned into pLV[Exp]-mUbe2i_LA:Insert_Y68F:mUbe2i_RA by Vector Builder (UBC9 donor lentiviral vector). Additionally, a scramble gRNA was designed and cloned into pLV[gRNA]-mCherry-U6 > Scramble_gRNA1 (Scramble) by vector builder. All of these were packed in an integrase-deficient lentivirus and purified by Vector Builder.

#### In vivo CRISPR gene editing

To mutate the in vivo UBC9 Y68 to F68 residue, we used the CRISPR-based genome editing strategy. Briefly, we divided 24 mice into two groups: 12 mice in WT (control) and 12 mice in HDR. Each group was further divided on the basis of the diet received: six mice each from WT and HDR, respectively, received a pair-fed diet, and six mice each from WT and HDR received an NIAAA diet. For lentivirus delivery, the mouse was placed in a restrainer, and 70% ethanol was applied to the tail to swell the vein slightly. The gRNA/Cas9 [pLV[Exp]-U6 > mUbe2i[gRNA#51] > CD68(short) > hCas9(ns):T2A:Puro], donor (pLV[Exp]-mUbe2i_LA:Insert_Y68F:mUbe2i_RA), and scramble (pLV[gRNA]-mCherry-U6 > Scramble_gRNA1) lentivirus particles were generated by Vector Builder as we described above. Donor UBC9 lentiviral vector (50 μl) mixed with 50 μl of scramble (WT) or gRNA/Cas9 (HDR) was injected into the tail vein of mice over 10 s, using a ^1^/_2_ inch (1.27-cm) 26-gauge needle and a 1-ml syringe on days 1, 5, and 9. On day 11, mice were euthanized 9 hours after gavage, followed by collection of blood and tissues, which were stored at −80°C. NGS confirmed the gene editing efficiency.

#### In vitro CRISPR gene editing

Lentivirus transduction in primary mouse KCs and RAW264.7 cells was performed according to the manufacturer’s instructions, and it was enhanced by polybrene (5 μg/ml; Vector Builder, PL0001). Briefly, 1 day before transduction, cells were seeded into six wells (3 × 10^5^ cells per well) and then cultured in DMEM, supplemented with 2 mM glutamine, 10% FBS, streptomycin sulfate (50 mg/ml), and 50 mM penicillin, in an incubator with 5% CO_2_ at 37°C overnight. Next, the original medium was replaced with 1 ml of DMEM (2 mM glutamine and 10% FBS) containing 2 × 10^6^ TU lentivirus and 5 μg of polybrene (Vector Builder, PL0001) for transduction. The cells were then incubated at 37°C overnight with 5% CO_2_. On day 2, the lentivirus-containing medium was replaced with 2 ml of fresh complete medium with or without LPS (500 ng/ml; Sigma-Aldrich, L3137-10MG) for overnight culture. To confirm successful transduction, mCherry signals were observed after 24 hours of transduction, under the BZ-X800 fluorescence microscope (Keyence, Osaka, Japan). After 48 hours of transduction, genomic DNA from cells was extracted and amplified by polymerase chain reaction (PCR) using two specific primers (table S1) to amplify the region around the *Ubc9* mutated site to detect HDR-mediated gene editing, which was confirmed by NGS. Additionally, culture medium was saved for coculture experiments at −80°C.

### Custom peptide synthesis

A custom d-amino acid phospho-peptide mimicking the phosphorylated tyrosine-68 (pY68) region of mouse UBC9 was prepared using solid-phase peptide synthesis by GenScript Biotech Corporation (Piscataway, NJ, USA). The final sequence was FITC–aminohexanoic acid (Ahx)–d-Arg–d-Met–d-Leu–d-Phe–d-Lys–d-Asp–d-Asp–phospho-d-Tyr–d-Pro–d-Ser–d-Ser–d-Pro–d-Pro–d-Lys–d-Cys–NH_2_. All amino acids were in d-stereoisomeric configuration to enhance proteolytic stability. The peptide was modified with a FITC group attached via an Ahx spacer at the N terminus to enable visualization in live-cell assays. C-terminal amidation increases structural stability and prevents degradation. Purity (>95%) was confirmed by HPLC and MS (data provided by the vendor). Peptide stocks were dissolved in sterile dimethyl sulfoxide at 1 mM and stored at −80°C in aliquots to avoid freeze-thaw cycles.

### In vitro transient transfection

#### Plasmid transfection

RAW264.7 cells were cultured in six-well plates (0.2 × 10^6^ cells per well) and transfected using 2 μl of JetPEI transfection reagent (PolyPlus, 101000053) with 1 μg of UBC9-HA tagged vector or empty vector. After 4 hours, the transfection medium was changed to a normal medium, and cells were cultured for 24 hours. Further, cells were processed for reverse transcription (RT)–PCR and microscopic analyses.

#### Peptide transfection

RAW264.7 cells were cultured in six-well plates (0.2 × 10^6^ cells per well) and transfected using 2.5 μl of ProteoJuice protein transfection reagent (Sigma-Aldrich, 71281-.125ML) with 5 μg of UBC9-pY68 peptide (phosphomimetic) for 2 hours followed by replacing the transfection medium with complete DMEM. After overnight incubation, RT-PCR and microscopic analyses were conducted.

### Phospho-peptide mapping

UBC9 was immunoprecipitated from primary hepatocytes or primary KCs using a UBC9 agarose-conjugated (AC) antibody from Santa Cruz Biotechnology (sc-271057-AC). The resulting UBC9 beads were sent to Applied Biomics (Hayward, CA, USA) for phospho-peptide mapping. Tryptic peptides were enriched for phospho-peptides, which were then analyzed by MS to identify phospho-sites. Phosphorylated residues were confirmed on the basis of MS data showing the neutral loss of phosphate, indicated by peak shifts in the MS/MS spectrum (Table 1). A 98-Da reduction in the observed mass of a phospho-peptide confirmed the neutral loss of phosphate from a single serine or threonine residue.

### Mass spectrometry

#### Affinity purification of UBC9-interacting proteins

Equal amounts of total liver protein (700 μg) were incubated overnight at 4°C with 40 μl of UBC9 agarose-conjugated antibody (Santa Cruz Biotechnology, sc-5231-AC) under gentle rotation. Immune complexes were washed five times with radioimmunoprecipitation assay (RIPA) buffer and two final times with phosphate-buffered saline (PBS). The UBC9-bound agarose beads were then collected and shipped to Applied Biomics (Hayward, CA, USA) for downstream MS analysis. Briefly, proteins bound to the UBC9 were subjected to in-gel digestion following reduction (5 mM dithiothreitol) and alkylation (15 mM indole-3-acetic acid). Peptides were analyzed by nanoLC-MS/MS using an Easy-nLC 1200 (Thermo Fisher Scientific) coupled to an Orbitrap Fusion Lumos (Thermo Fisher Scientific). MS1 spectra were acquired at 120,000 resolution, followed by higher-energy collisional dissociation (HCD) fragmentation (collision energy of 30) for MS2 scans at 15,000 resolution.

#### Data analysis and interaction mapping

Raw MS data were processed at Applied Biomics using MaxQuant for LFQ. Protein identification was performed against the UniProt human database, with a false discovery rate of <1%. Background contaminants and nonspecific interactors were filtered using SAINTexpress, comparing UBC9 pulldowns with immunoglobulin G (IgG) controls. The processed proteomics data have been deposited in Zenodo under the DOI: 10.5281/zenodo.15313911. Functional interaction between kinases and UBC9 was analyzed.

### Generation of pUBC9 antibody

To investigate the role of pUBC9 in ALD, a custom phospho-specific UBC9 antibody (RRID: AB_3695637) was generated by ABclonal Technology (Woburn, MA, USA). The antibody was designed to recognize UBC9 phosphorylated at tyrosine-68 (Y68) specifically. Antibody generation, purification, and validation are described in Supplementary Methods.

### Western blotting

The liver tissues and harvested cells were lysed with RIPA buffer containing a cocktail of protease and phosphatase inhibitors (1:100; Thermo Fisher Scientific, 78442) on ice for 30 min, and the protein concentration was determined using the Bradford protein assay (Bio-Rad, 5000006) after centrifugation. After electrophoresis in a 10% SDS–polyacrylamide gel electrophoresis (PAGE) gel, proteins were transferred to nitrocellulose membranes (NCM) (Sigma-Aldrich, GE10600002) and blocked in 5% skin milk (Apex, 20-241) for 1 hour and then incubated with primary antibody (table S2) at 4°C overnight. The membranes were then incubated with horseradish peroxidase–conjugated antibody (1:8000, table S2) for 1.5 hours. Immunoreactive bands were detected with a Radiance ECL kit (Azure Biosystems, AC2204) or Amersham ECL Prime Western Blotting Detection Reagent (Cytiva, RPN2235) according to the manufacturer’s instructions. All blots were analyzed by ImageJ software (versions 1.51 and 1.54j) for quantification.

### Phos-tag analysis

Phos-tag acrylamide (Wako Chemicals, AAL-107 M) selectively binds to phosphorylated proteins, modifying their mobility on SDS-PAGE, as outlined in our previous study (*10*). Briefly, extracted proteins from primary KCs or RAW264.7 cell extracts were separated on SDS-PAGE gels (10%) containing manganese chloride and 15 μM phos-tag, following established protocols. Western blotting was conducted with the UBC9 antibody as described earlier.

### Immunoprecipitation

Immunoprecipitation analysis involved precleaning 700 μg of whole-cell extract with mouse IgG-AC (Santa Cruz Biotechnology, sc-2343) for 30 min at 4°C under rotation. Immunoprecipitation of extracted proteins was performed using 3 μg of anti-UBC9 antibody overnight at 4°C under rotation. To pull down the protein-antibody complexes, the latter was incubated with protein A/G-magnetic beads (40 μl; Thermo Fisher Scientific, 88803) for 1 hour at 4°C under rotation before washing the conjugate five times with 1 ml of incubation buffer [150 mM NaCl, 1 mM EDTA, 1 mM EGTA (pH 8.0), 50 mM tris-Cl (pH 7.5), 1% (v/v) Nonidet P-40 (10%, v/v), and 25 mM NaF]. Further, 10% SDS-PAGE was used to separate the immunoprecipitated proteins for Western blotting, and the blot was probed with previously described antibodies. VeriBlot (Abcam, ab131366) was used to minimize background noise, and normal IgG (Santa Cruz Biotechnology, sc-2025) was used as a control.

### In vitro kinase assay

SRC kinase activity was assessed in a kinase reaction buffer containing 2 μl each of MgCl_2_ (50 mM) and dithiothreitol (10 mM), along with 100 μM ATP, 120 ng of SRC kinase (Active Motif, 81115), and 1 μg of the substrate (UBC9) (Prospec, ENZ-341) at 37°C for 1 hour. The reaction was terminated by adding 4 μl of sample loading buffer and 11 μl of RIPA buffer, followed by denaturation at 95°C for 5 min. The kinase activity was detected by Western blotting using the appropriate antibodies (table S2).

### Real-time RT-PCR

Total RNA was isolated by the Quick-RNA Miniprep Kit (Zymo Research, D4203-A) and reverse transcribed with a cDNA reverse transcription kit (Lucigen, F83902-1) according to the manufacturer’s instructions. Quantitative RT-PCR quantified the mRNA levels with iTaq Universal (Bio-Rad, 1724134). We normalized the relative mRNA levels against *Gapdh* mRNA levels. The probes used for RT-PCR are provided in table S3.

### BODIPY staining in vitro

After ethanol treatment of hepatocytes from coculture experiments as previously described, cells were washed with PBS and fixed in 4% paraformaldehyde for 15 min at room temperature. Hepatocytes were stained with 2 μM BODIPY (Invitrogen, D3922) in PBS for 15 min at 37°C in the dark. After washing with PBS twice, the nuclei were counterstained with 4′,6-diamidino-2-phenylindole (DAPI). Image acquisition was conducted using the BZ-X800 fluorescence microscope (Keyence, Osaka, Japan).

### Duolink PLA

PLA was used to detect the interaction of UBC9 with pTyr and SRC in paraffin-embedded human alcoholic steatohepatitis liver tissue microarray (Xenotech, TMA.ASH lot no. 2210241) and NIAAA mouse liver tissue sections. Specifically, after deparaffinization, antigen retrieval, and permeabilization, Duolink PLA probe anti-rabbit PLUS (MilliporeSigma, DUO92002) and Duolink PLA probe anti-mouse MINUS (MilliporeSigma, DUO92004) were used according to the manufacturer’s instructions with some modifications to process the tissue sections. Briefly, tissues were incubated with primary UBC9 (rabbit; Abcam, ab33044) and pTyr (mouse; Cell Signaling Technology, 9411S) or SRC (mouse; Abcam, ab231081) antibodies (1:150) overnight at 4°C in a humidity chamber after blocking. Then, tissues were incubated with PLUS as well as MINUS PLA probes (1:250) overnight at 4°C in a humidity chamber, and Duolink in situ detection reagent kit (MilliporeSigma, DUO92008-red) was used according to the manufacturer’s instructions with slight modifications up to the amplification step to detect protein interaction. Further, to detect the site of these protein interactions, tissue sections were incubated with anti-F4/80 antibody (1:100; Sigma-Aldrich, SAB5500103) overnight at 4°C in a humidity chamber. Next, tissue sections were washed three times with wash buffer A and incubated with secondary antibody (1:200; Abcam, ab150077). Tissue sections were counterstained with Vectashield mounting medium with DAPI (Vector Laboratories, H-1200-10) and observed under the BZ-X800 fluorescence microscope (Keyence, Osaka, Japan), where the PLA signal was recognized as a red fluorescent spot. Last, PLA fluorescence dots were quantified by ImageJ.

### Immunofluorescence microscopy

To evaluate the localization of p65, mouse liver tissue sections from all groups were deparaffinized, and the antigen was retrieved using the Universal HIER antigen retrieval reagent (ab208572, Abcam, Cambridge, UK). After permeabilization with PBS containing 0.5% Triton X-100 and blocking with 5% goat serum prepared in PBS containing 0.1% Tween, tissue sections were incubated with 1:100 diluted anti-p65 antibody (Proteintech, 10745-1-AP) (table S2) overnight in a humidity chamber at 4°C. Afterward, the tissue sections were washed with PBS containing 0.1% Tween and incubated with secondary antibody (1:200, rabbit, Alexa Fluor 594; Abcam, ab150080) (table S2) for 1 hour at room temperature. Last, tissues were again washed with PBS containing 0.1% Tween before counterstaining with Vectashield mounting medium with DAPI (Vector Laboratories, H-1200-10) and visualized under a fluorescence microscope (BZ-X800, Keyence, Osaka, Japan). For the negative control, no primary antibody was added (fig. S7).

### Histopathological analysis

Liver specimens were fixed with 4% paraformaldehyde overnight, followed by embedding in optimal cutting temperature (OCT) compound or paraffin. The paraffin blocks were then sectioned into 5-μm-thick slices and stained with hematoxylin and eosin (Abcam, ab220365) to assess liver injury. In contrast, the OCT sections were used for Oil Red O (Sigma-Aldrich, 01516), or BODIPY (Invitrogen, D3922) staining. Images were captured with the BZ-X Series All-in-One Fluorescence Microscope (Keyence, USA).

### Biochemistry and cytokine assays

Plasma ALT and AST levels from mice were measured using the ALT (MET-5123) and AST (MET-5127) colorimetric activity assay kits from Cell Biolabs (San Diego, CA, USA). TNF-α (KE10002), IL-1β (KE10003), and IL-6 (KE10007) levels were assessed by enzyme-linked immunosorbent assay kits from Proteintech (Rosemont, IL, USA). The triglyceride levels in liver tissue homogenates and PMHs were detected using the Triglyceride Colorimetric Assay Kit (Cell Biolabs, STA-396) according to the manufacturer’s protocols.

### Chromatin immunoprecipitation

The ChIP assay was conducted using the ChIP-IT Express Enzymatic Chromatin Immunoprecipitation Kits (Active Motif, 53009) according to the manufacturer’s instructions. Liver tissues (50 mg) were washed with PBS and treated with formaldehyde for cross-linking. Immunoprecipitation was performed with the anti-p65 antibody (Active Motif, 39369) and rabbit IgG, followed by extraction of DNA with chloroform. PCR analyzed precipitated DNA. The primers used for PCR are provided in table S4. PCR products with 267–base pair (bp) and 421 bp for Cebpβ and SrebF1 promoters were lastly examined by electrophoresis and gel extraction.

### Statistical analysis

Data are expressed as means ± SEM. Statistical analysis was conducted using Student’s *t* tests, analysis of variance (ANOVA), and Fisher’s tests, and statistical significance was defined as *P* < 0.05. For mRNA and protein levels, the ratios of gene and protein expression to the respective housekeeping densitometric values were compared. In vitro experiments were performed at least three times, independently, and four to five mice per group for in vivo experiments. The primary hepatocytes for in vitro experiments were obtained from the livers of three independent mice. Densitometry analyses were performed using ImageJ Software (version 1.52q), and calculations were performed using GraphPad Prism (version 9.0.0) and Microsoft Excel (version 16.54).

## Supplementary Materials

### This PDF file includes:

Supplementary Methods Figs. S1 to S7 Tables S1 to S6 Raw data References
