# Supplementary material for: SRC-mediated phosphorylation of UBC9 regulates inflammatory and metabolic signaling in alcohol-associated liver disease
Source: Sci Adv. 2026 Apr 10;12(15):eaec0138. doi: 10.1126/sciadv.aec0138 (PMC13068051; doi:10.1126/sciadv.aec0138)
Supplement: Supplementary file 1 — Supplementary Methods Figs. S1 to S7 Tables S1 to S6 Raw data References [file sciadv.aec0138_sm.pdf]

Science Advances
AAAS

Supplementary Materials for

SRC-mediated phosphorylation of UBC9 regulates inflammatory and metabolic signaling in alcohol-associated liver disease

Swati Chandla *et al.*

Corresponding author: Maria Lauda Tomasi, marialauda.tomasi@cshs.org; Komal Ramani, komal.ramani@cshs.org

Sci. Adv. **12**, eaec0138 (2026)
DOI: 10.1126/sciadv.aec0138

This PDF file includes:

Supplementary Methods
Figs. S1 to S7
Tables S1 to S6
Raw data
References

SUPPLEMENTARY METHODS

Animal studies-sex as a biological variable

Our prior experiments in alcohol-fed mice showed similar responses to UBC9 and other molecular players we assessed between male and female mice (*12, 21*). Sex-specific differences between male and female mice in terms of alcohol feeding are known with female mice showing more susceptibility to alcohol-induced liver damage but less alcohol-heightened aggression compared to male counterparts (*40, 41*). Therefore, in this study female mice were used.

Generation of phosphorylated UBC9 antibody

To investigate the role of phosphorylated UBC9 in ALD, a custom phospho-specific UBC9 antibody (RRID: AB_3695637) was generated by ABclonal Technology (Wuhan, China). The antibody was designed to specifically recognize UBC9 phosphorylated at tyrosine 68 (Y68). The process involved synthesizing a modified antigen peptide, FKDD(Y-p)PSSP-C, corresponding to the phosphorylated form of UBC9, along with its non-phosphorylated counterpart, FKDDYPSSP-C. Peptides were synthesized with a purity of ≥85% and conjugated to keyhole limpet hemocyanin (KLH) for immunization. Three New Zealand white rabbits were immunized with the phospho-peptide as following: initial immunization (0.70 mg with complete Freund's adjuvant) followed by four booster doses (0.35 mg each with incomplete Freund's adjuvant) at defined intervals. Serum samples were collected after the final immunization and screened for phospho-specific reactivity using dot-blot assays against both modified and non-modified FKDDYPSSP peptides. Affinity purification of the phospho-UBC9 antibody was done using antigen affinity chromatography. To ensure specificity, sera were first passed through a modified-peptide column to enrich phospho-UBC9 antibodies, followed by a second step using a non-modified-peptide column to remove antibodies recognizing the unphosphorylated form. Purified phospho-UBC9 antibodies (E25961(P), E25962(P), and E25963(P)) were tested

via dot-blot assays, demonstrating high specificity and sensitivity for phosphorylated UBC9. Finally, antibody preparations were stored in PBS (pH 7.3) with 50% glycerol and 0.05% Proclin 300 and validated for use in Western blotting at dilutions of 1:500–1:1000.

Fig. S1

A.

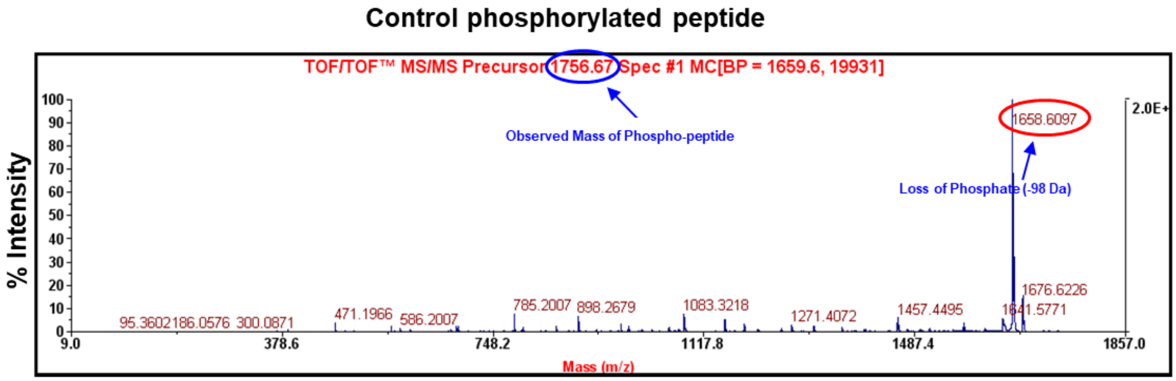

B.

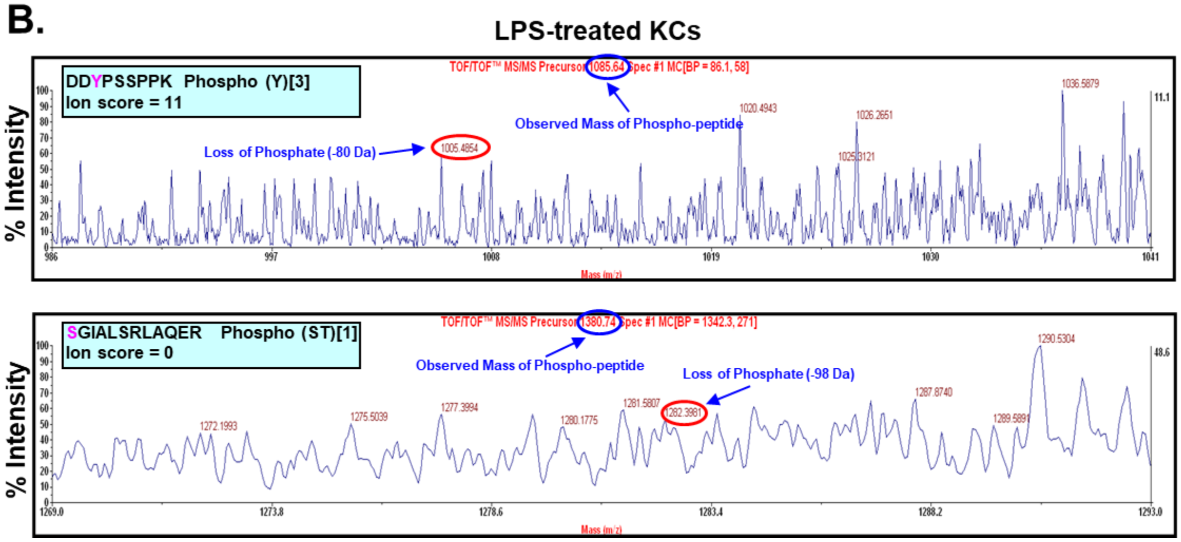

Fig. S1. MS/MS spectral analysis of phospho-peptides mapped for UBC9. The spectral analysis of DDYPSSPPK peptide for KCs treated with LPS as summarized in Table 1. (**A**) Synthetic control peptide corresponding to the expected phosphorylated fragment TOF/TOF™ MS/MS precursor mass = 1756.67 Da. A neutral loss of phosphate (-98 Da) from the precursor ion. (**B**) MS/MS analysis of peptides from cells treated with LPS identified an endogenous peptide that shows a neutral loss of phosphate from tyrosine (Y) causes an 80 Da decrease in the mass of the precursor peptide.

Fig. S2

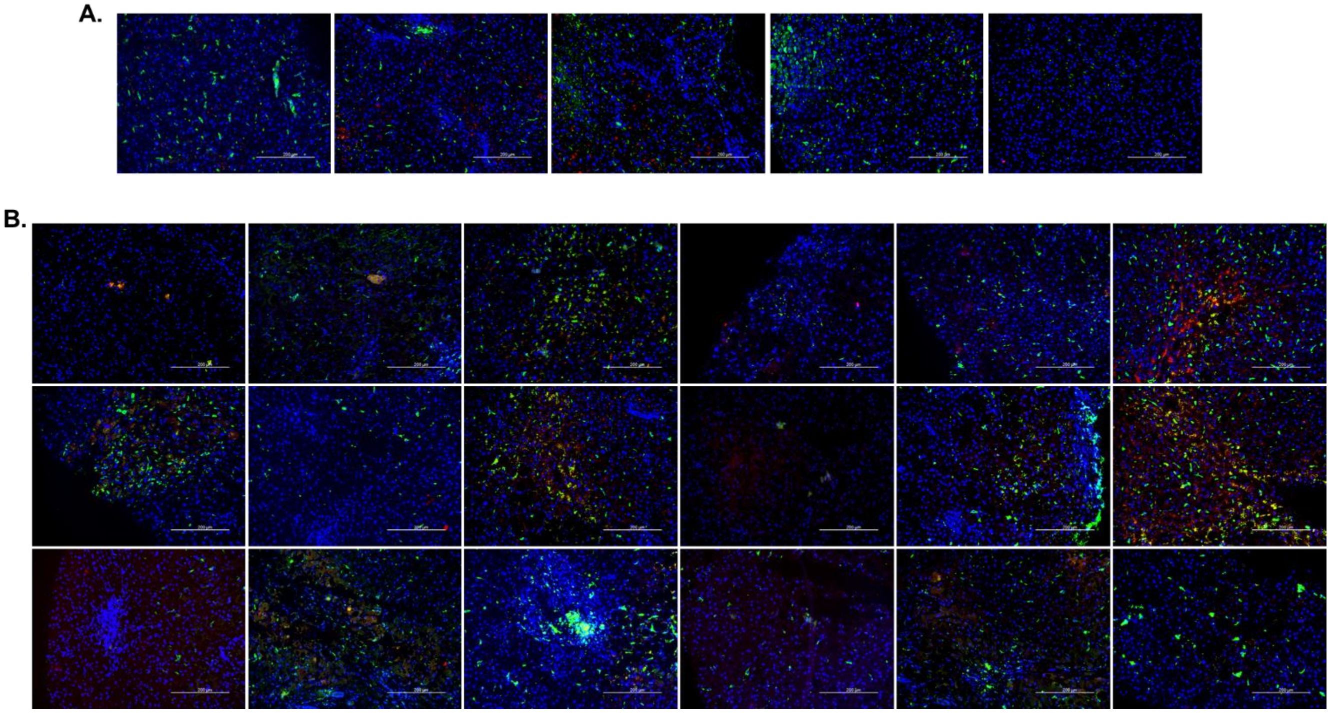

Fig. S2. UBC9 phosphorylation induced in KCs upon alcohol exposure. Proximity ligation assay (PLA) demonstrating increased pUBC9 signal in $CD68^{+}$ macrophages in (**A**) human normal livers (n=5) and (**B**) alcoholic steatohepatitis livers (ASH, n=18). Scale bar, 200µm.

Fig. S3

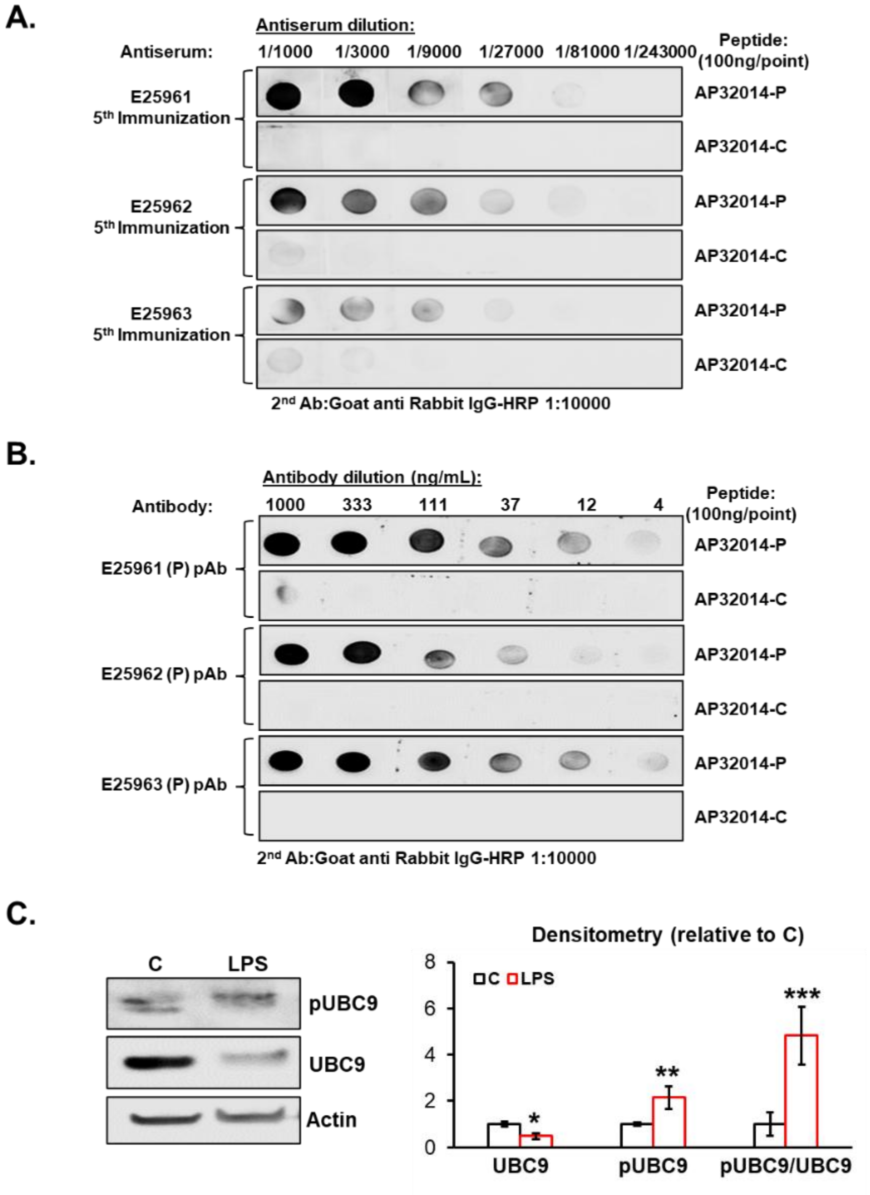

Fig. S3. Custom anti-pUBC9Y68 antibody efficiently recognized phosphorylated UBC9 in RAW264.7 cells treated with LPS. (**A**) Selected antiserums (E25961; E25962; E25963) against unphosphorylated (AP32014-C) and phosphorylated (AP32014-P) UBC9 peptides were tested by western blot binding assay. (**B**) Serial dilutions of purified antibodies against AP32014 peptides were analyzed as above. (**C**) RAW264.7 cells were treated with LPS (500ng/mL for 16h). Protein level of phosphorylated UBC9 at Y68

residue was measured by western blot using the custom pUBC9Y68 antibody (E25961). A goat anti-rabbit HDR-conjugated antibody was used as 2nd antibody (dilution, 1:10,000), while actin was used as housekeeping. Ratio of total UBC9 vs pUBC9Y68 was measured by ImageJ. Mean ± SE (n=4). *p<0.01 vs control UBC9; **p<0.03 vs control pUBC9; ***p<0.02 vs control pUBC9/UBC9.

Fig. S4

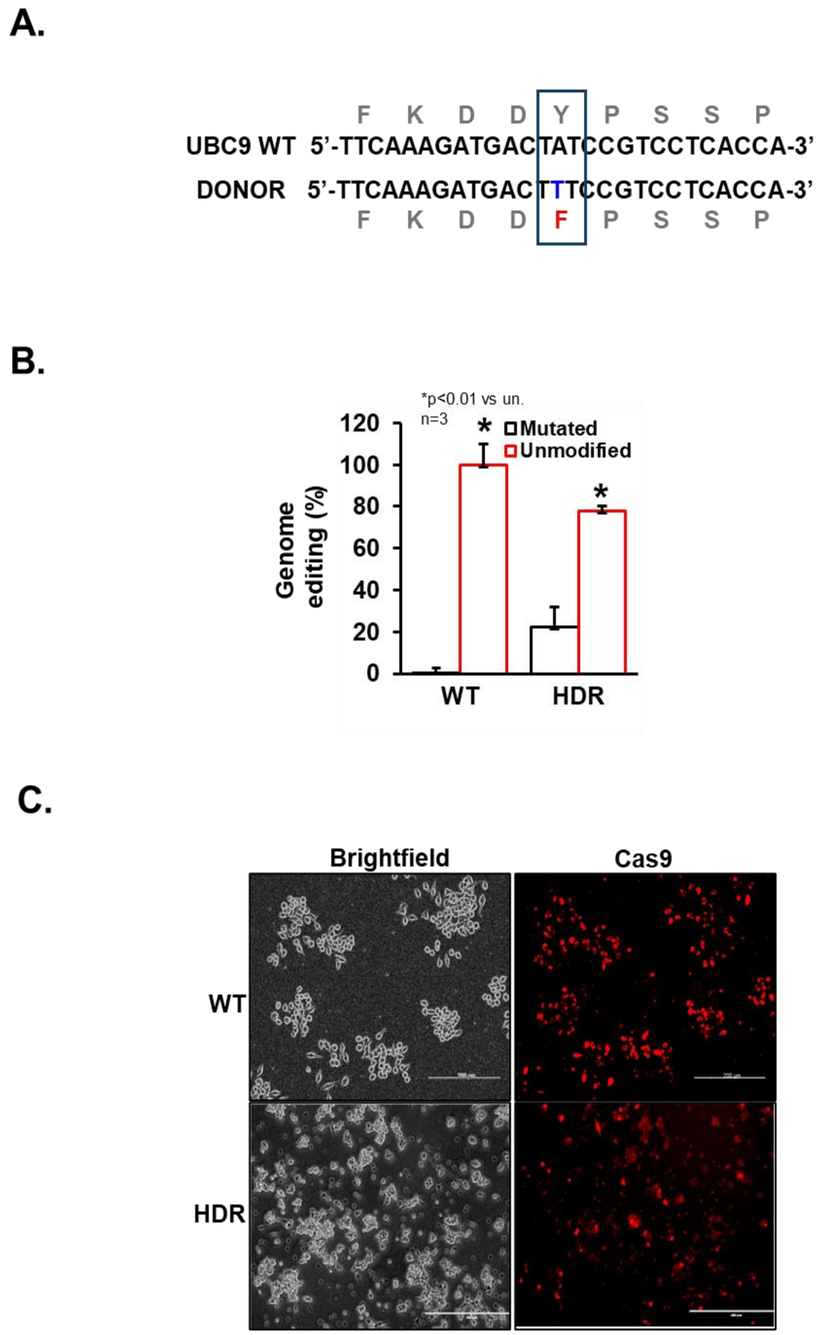

Fig. S4. CRISPR/Cas9-mediated gene editing of UBC9 Y68 to a non-phosphorylatable phenylalanine (Y68F). RAW264.7 cells were CRISPR gene edited as indicated in Methods section. (**A**) Schematic representation of CRISPR/Cas9-mediated mutation of UBC9 Y68 to F68. (**B**) Gene editing efficiency was tested by NGS. Mean ± SE (n=3). *p<0.01 vs unmodified. (**C**) The transduction efficiency was verified by immunofluorescence of Cas9 (red) (200μm) (20X).

Fig. S5

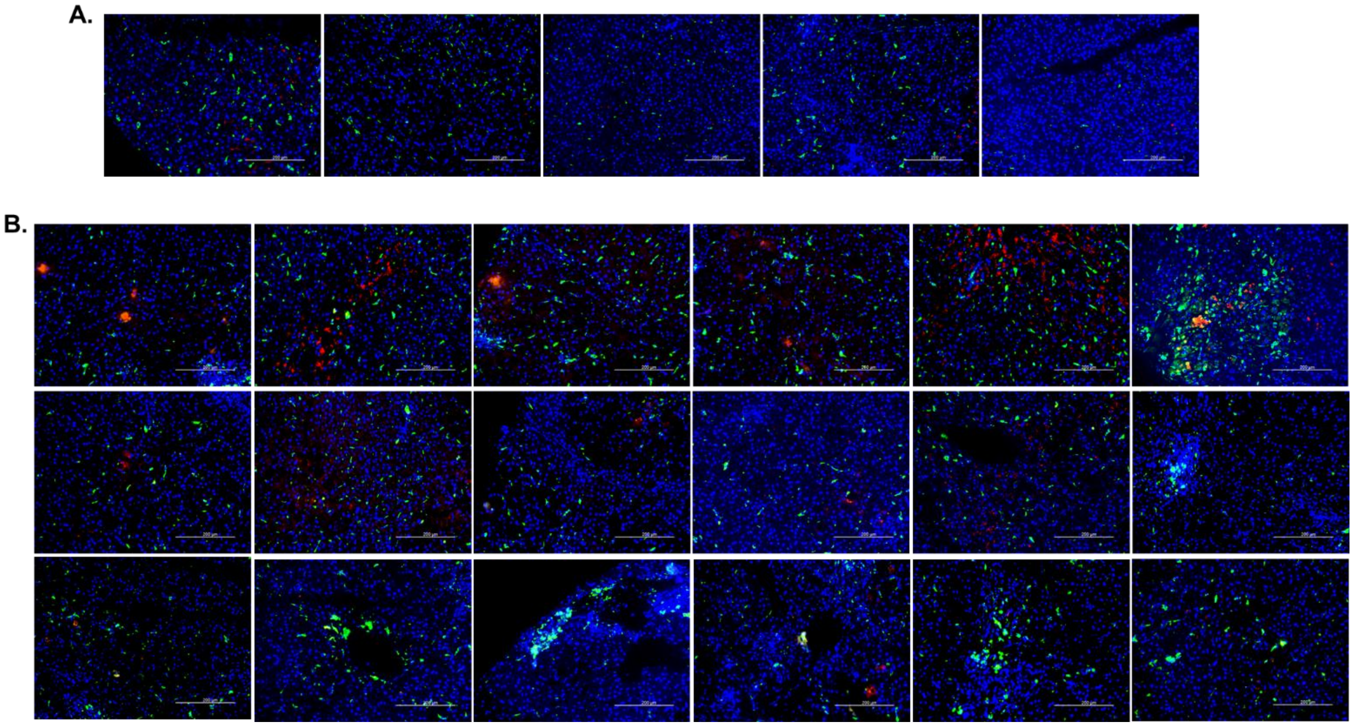

Fig. S5. UBC9 interacts with SRC in human ALD. Proximity ligation assay (PLA) detection of UBC9 interacting with SRC (red) in CD68+ KCs (green) in (A) human normal livers (n=5) and (B) alcoholic steatohepatitis livers (ASH, n=18). Scale bar, 200μm.

Fig.S6

A.

```
Mouse Srebf1 chr11:60218594-60222594

>FP015465 Srebf1_1 :+U EU:NC; range -1000 to 100.
ttttcagtctttattcaggcacctggcctggccccaggtcacatatgacccagtagctgg
gctcaggtcctcaggacctctgttacagaattctttctttttttttctttttttcttctctc
ttctcttctcttctcttctttctttttggtatcttgagacaaggtttctttgtattgccc
aggctgtcctgaaactcactctgtagatcatgctggcttcgaactcagaaacctacctgc
ttctgccacccaagtgctgggattaacagagtgtgccaccatgcctggctcacagaatcc
cttcttgactttgagattcacactttttctagatccttttggagacacaagtcattctat
aggagtagtacgagcccactggcccttcaggtcctctctgctccaatggtaggactgata
ggattaagacaagatcagaagttccagccacccccctcctcctgcaggttctgcctgagaa
ctcttctcctctggaggcccacttctagacttctaagctgagagggtggacaggtcttat
gggccatgcttctctaacctctcagctggctcatagataattggaccctggctacagatg
aggaaaacagaggcacatagtggaggggccccactgcaggcctgtatctgctgtatttg
gacccaggactgtctggttctaaacagcaagactaggaagtgagttttctgttctctgct
cccaccctcatgctgggccccccatccctggccctttaatctaacgatgtctgggctggg
tctagtcctgtggcttccattcggtccctcccttggcctgatacagaagaagccaaggcc
cagtgcctgtgcacccgcaggagattgctagaaaccaggctcgggcaggcagtctcaacc
cgctaggcgagctgtcaggatgcaggctggtgggcggggcttgatgggaaaccccgcccc
tccgctcaggtggctccgcccgcggaacccagtttccgggGAACTTTTCCTTAACGTGGG
CCTAGTCCGAAGCCGGGTGGGCGCCGGCGCCATGGACGAGCTGGCCTTCGGTGAGGCGGC
TCTGGAACAGACACTGGCCGA

tgggaaacccc -56/-45 p65 binding motif #1

gttttctgttc -297/286 p65 binding motif #2
```

B.

```
Mouse Cebpb chr2:167686966-167690966

>FP003358 Cebpb_1 :+U EU:NC; range -800 to 100.
agcctctgggcagggctgtgcaggtcttaggaaggcatcgtacttctcggggggtccgttt
gtctctgatgacaccactactaacaataccaacactgtccacggagggaactcagaagca
aagtgactcacccagacacagtgtggcagaacccccagccctgccaccttcatggagagg
cgcagggagtggcgtaggcaggtgcggtaggcgggccatacatagggtggagggctggga
tccaggacagcgagcatccccggggtgggcgtgcacctggagagttctgcttcccaggag
ttgggggggggtcccagaggtgtgggcagcacctgggtgggaggacatgcaccccgtgg
aaggctgcttccagggaattggggcccggggacagcgggcaggatccgagggttggggtc
gtgtagctggaggaacgatctgtttcccaagagttgggggtccgggacagcggaccggag
ccgggacgagccaccgcgtcctccagcaggggcgggcggcggccgcgctcctccgtgtt
cccgaggggcgggaagggggcgggctggcgtcacccgcgtccgtggccatcgcggggctg
gcgggacgcccgggtcaccgctcagcctcccgcgcgaggacccccgccgcgccccactcc
ccgccgcgccctctcgcgctcccgtggccgggcaatgacgcgcaccgaccgggtggcggg
gcggcgggagggccccagcgtgacgcagcccgttgccaggcgccgccttataaacctcc
cgctcggccgccgccgcgccGAGTCCGAGCCGCGCACGGACCGGACGCAGCGGAGCCC
GCGGGCCCCGCGTTCATGCACCGCCTGCTGGCCTGGGACGCAGCATGCCTCCCGCCGCCG
C

gggggtccc -493/-484 p65 binding motif #1

gggaactca -695/-686 p65 binding motif #2
```

Fig. S6. P65 binding motifs in the promoter regions of murine SrebF1 and Cebpβ. (**A**) Putative p65 binding motifs of mouse SrebF1 promoter (-1000 bp to +100 bp relative to the transcription start site). The first motif (tgggaaaccc) is located at -56/-45, while the second motif (gttttcctgtct) is located at -297/-286) highlighted in yellow. (**B**) Putative p65 binding motifs of mouse Cepbβ promoter (-800 bp to +100 bp relative to the transcription

start site). The first motif (gggggtccc) is located at -493/-484, while the second motif (gggaactca) is located at -695/-686) highlighted in yellow.

Fig. S7

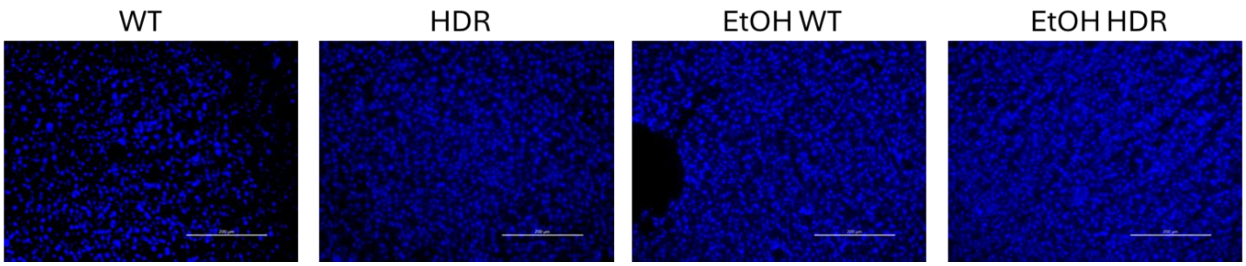

Fig. S7. Supplementary to Immunofluorescence of p65 (red) in liver sections. Microscopic images showing negative controls for each group. Nuclei were stained with DAPI (blue). Scale bar, 200μm.

Table S1. List of primers used for NGS-PCR

Gene	Supplier	Sequence (5'-3')
Ube2i (Ubc9)	Azenta/Genewiz, South Plainfield, NJ, USA	Fw: GCTCAGTTGCAGAGTATGC
		Rev: CATCTACCCTGGGACTTGT

Table S2. List of antibodies used for Western Blot.

Product	Catalog #	Supplier	Application
UBC9	AB33044	Abcam, Cambridge, UK	WB, IF, IP and PLA
pUBC9	E25961	ABclonal Technology, Woburn, MA, USA	WB
SREBP1C	66875-1-Ig	Proteintech, Rosemont, IL, USA	WB

CEBPβ	3082S	Cell Signaling, Danvers, MA, USA	WB
TNF-α	17590-1-AP	Proteintech, Rosemont, IL, USA	WB
pSRC (Tyr416)	6943S	Cell Signaling, Danvers, MA, USA	WB
SRC	2123S	Cell Signaling, Danvers, MA, USA	WB
pPKR (Thr446)	GTX132789	Genetex, Irvine, CA, USA	WB
PKR	GTX130941	Genetex, Irvine, CA, USA	WB
pGSK3β (Ser9)	67558-1-Ig	Proteintech, Rosemont, IL, USA	WB
GSK3β	22104-1-AP	Proteintech, Rosemont, IL, USA	WB
CLK4	OASG01649	Aviva Systems Biology, San Diego, CA, USA	WB
Anti-β-Actin−Peroxidase	A3854	Sigma-Aldrich, St. Louis, MO, USA	WB
Cas9	14697S	Cell Signaling, Danvers, MA, USA	IF
F4/80	SAB5500103	Sigma-Aldrich, St. Louis, MO, USA	IF, PLA
CD68	MA5-13324	Thermo-Fisher, Waltham, MA, USA	PLA
P65	10745-1-AP	Proteintech, Rosemont, IL, USA	IF
HRP-conjugated (mouse)	7074S	Cell Signaling, Danvers, MA, USA	WB
HRP-conjugated (rabbit)	7076S	Cell Signaling, Danvers, MA, USA	WB
Mouse Alexa fluor 488	ab150117	Abcam, Cambridge, UK	IF
Mouse Alexa fluor 594	ab150116	Abcam, Cambridge, UK	IF
Rabbit Alexa fluor 594	ab150080	Abcam, Cambridge, UK	IF
Rabbit Alexa fluor 488	ab150077	Abcam, Cambridge, UK	IF
Veriblot	ab131366	Abcam, Cambridge, UK	IP

Phospho-Tyrosine Mouse mAb (P-Tyr-100)	9411S	Cell Signaling, Danvers, MA, USA	PLA
Anti-Src antibody [GD11]	ab231081	Abcam, Cambridge, UK	PLA
normal IgG	sc-2025	Santa Cruz Biotechnology, Dallas, TX, USA	IP
UBC9 agarose-conjugated (AC) antibody	sc-271057-AC	Santa Cruz Biotechnology, Dallas, TX, USA	Phospho-peptide mapping

Table S3. List of probes used for qRT-PCR.

Gene	Catalog #	Supplier
Ube2i (Ubc9)	Mm00495850_m1	Thermo-Fisher, Waltham, MA, USA
Fasn	Mm00662319_m1	
Acc1	Mm01304258_m1	
Srebf1	Mm00550338_m1	
Cebpβ	Mm00786711_s1	
18S	Mm02601777_g1	
Tnf-α	Mm00443258_m1	
Il-6	Mm00446190_m1	
Il-1β	Mm00434228_m1	

Table S4. List of primers used for ChIP

Gene	Sequence (5'-3')	
	p65 binding motif #1	p65 binding motif #2
*Srebf1**	Fw: ATGAGGAAAACAGAGGCACA	Fw: TCTAGTCCTGTGGCTTCCATTC
	Rev: GAATGGAAGCCACAGGACTAGA	Rev: GGAAAAGTTCCCCGGAAAC
*Cebpβ**	Fw: GCTGTGCAGGTCTTAGGAA	Fw: AGCCCTCCACCCTATGTAT
	Rev: ATACATAGGGTGGAGGGCT	Rev: AGATCGTTCCTCCAGCTACAC

*primers were purchased from Azenta/Genewiz, South Plainfield, NJ, USA.

Table S5. List of Cell lines

Name	Citation	Supplier	Cat no.	Passage no.
Mouse: primary mouse hepatocytes	PMID:38100286	This paper	N/A	N/A
Mouse: primary mouse Kupffer Cells	PMID:27561301	This paper	N/A	N/A
RAW 264.7	PMID:40396606<br>PMID:40400000	American Type Culture Collection (Manassas, VA, USA)	TIB-71	Passage 4
THP1	PMID:40408638<br>PMID:40397754	American Type Culture Collection (Manassas, VA, USA)	TIB-202	Passage 4

Table S6. Human Tissue samples

Description	Source	Identifier
Human Liver	Xenotech, Kansas City, KS, USA	TMA.ASH lot no. 2210241

Raw Data

Figure 1

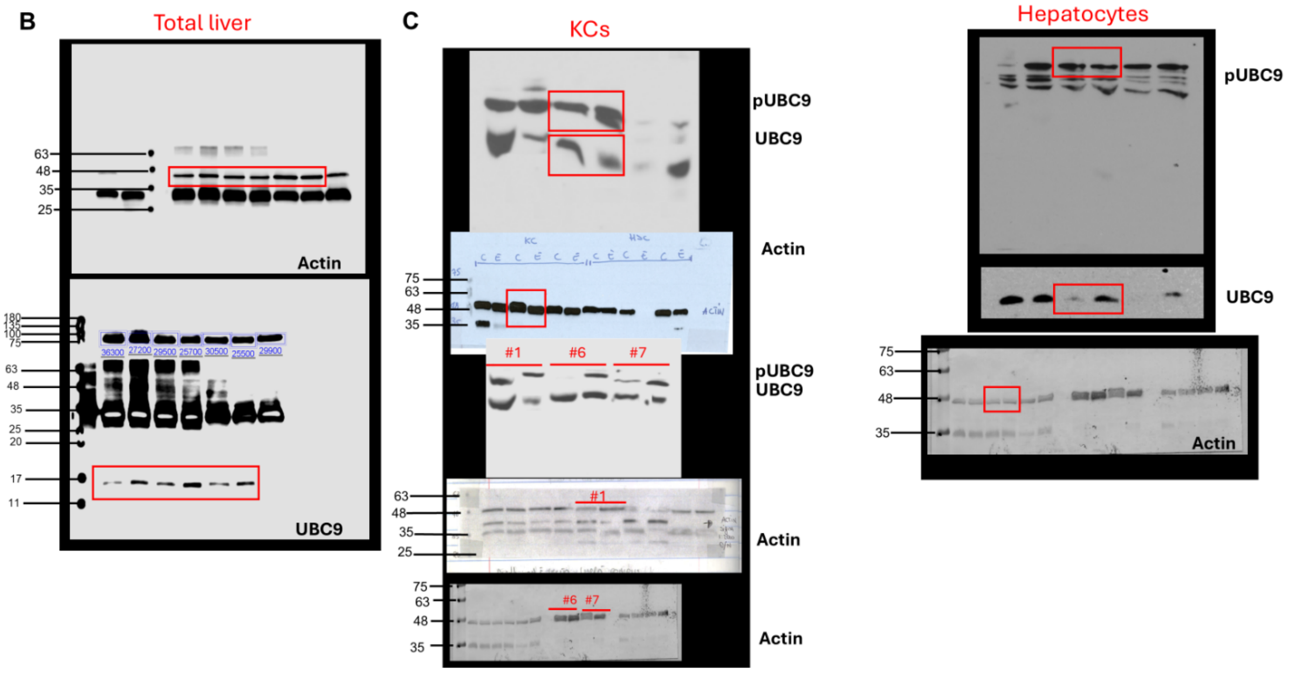

Figure 1D

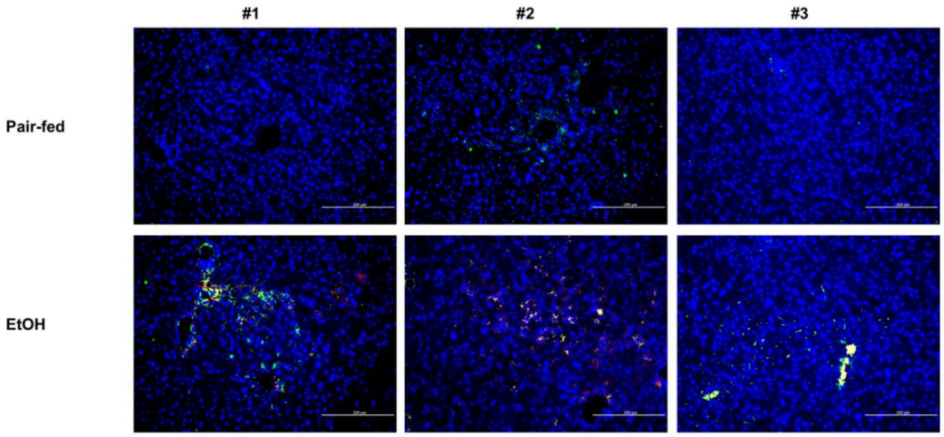

Figure 2

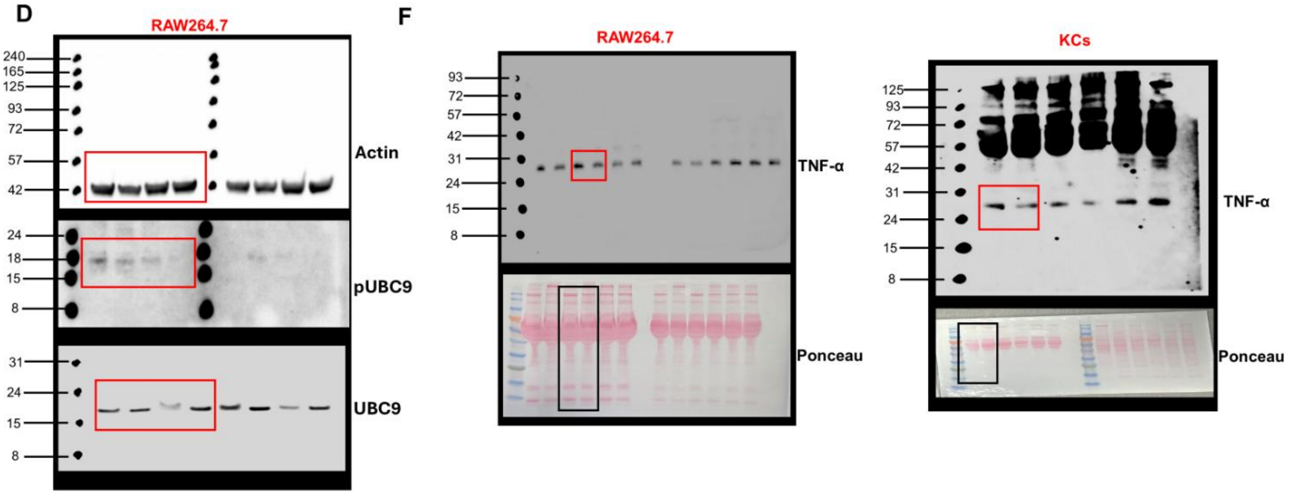

Figure 3A

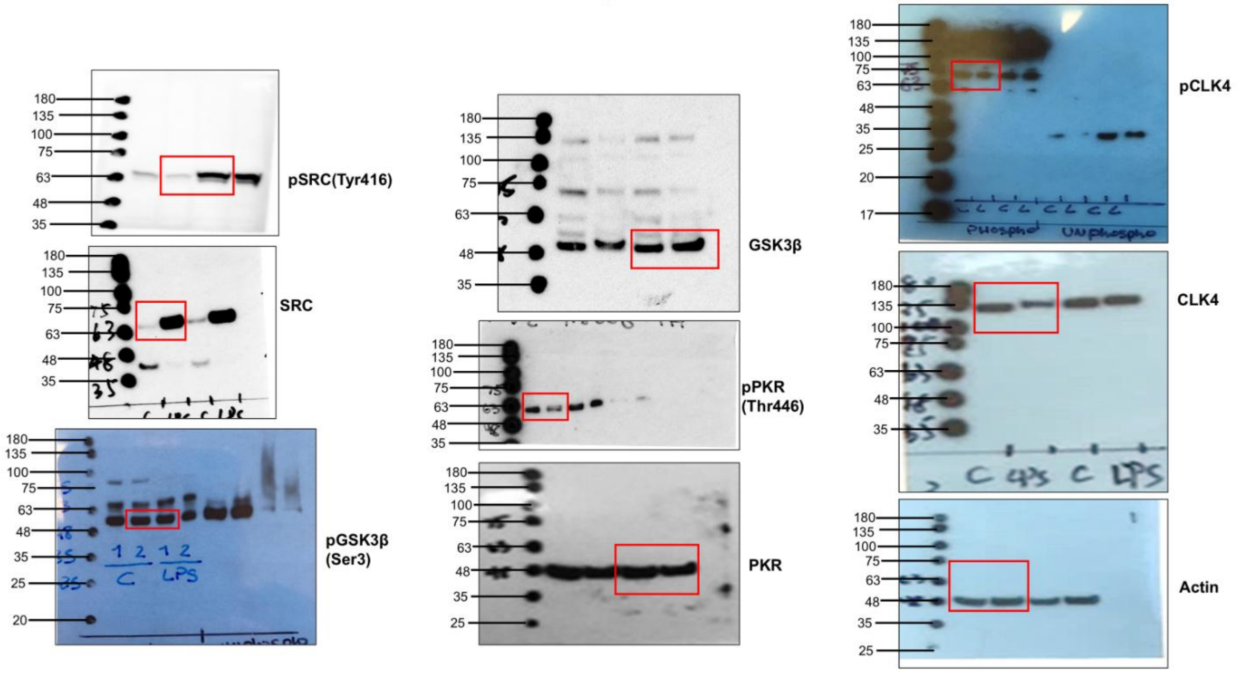

Figure 3D-E

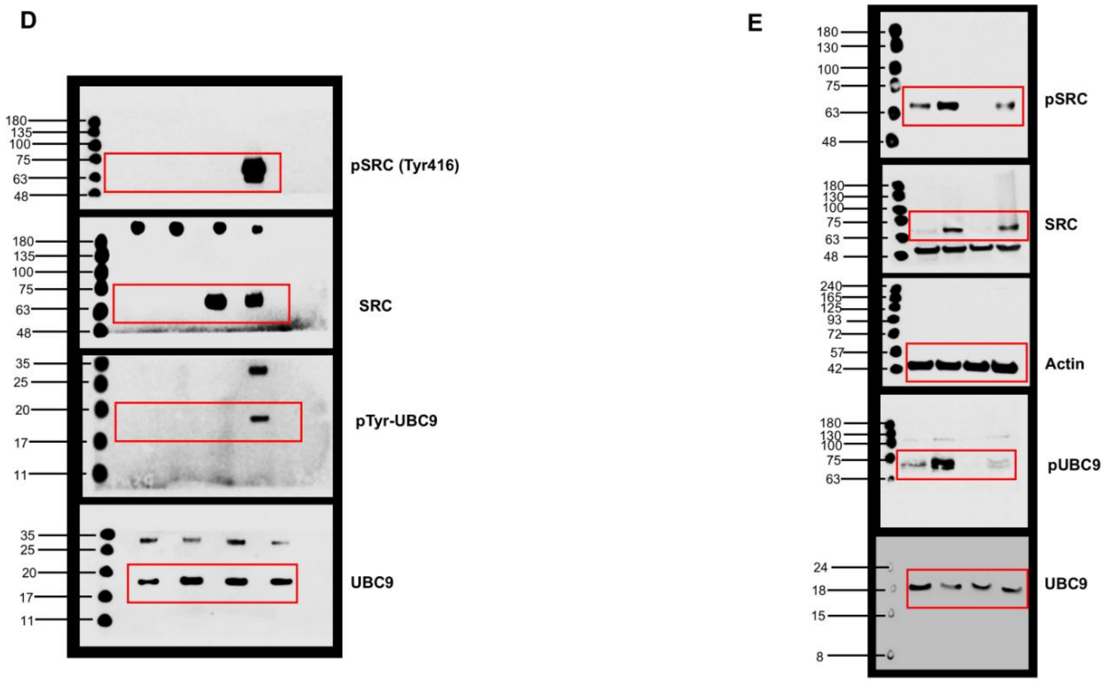

Figure 4

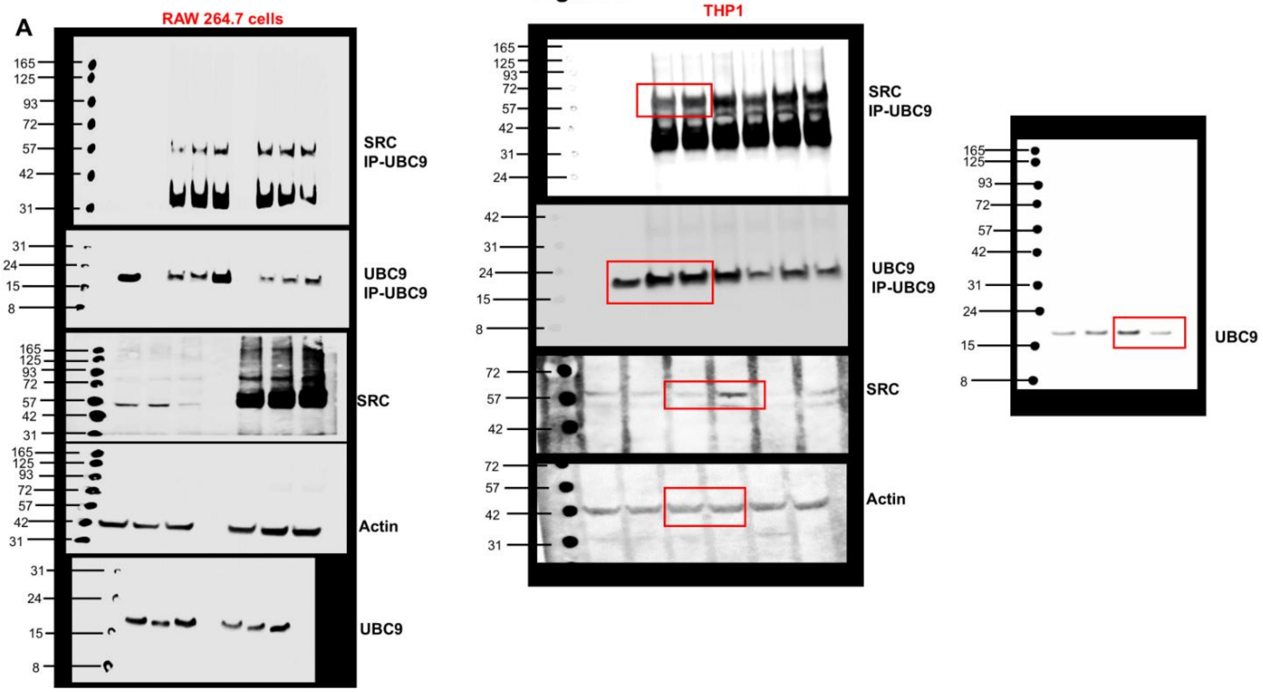

Figure 4B

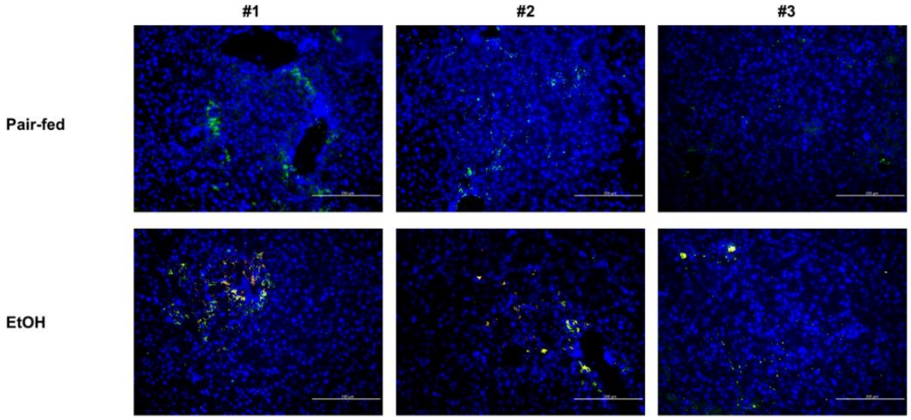

Figure 5A

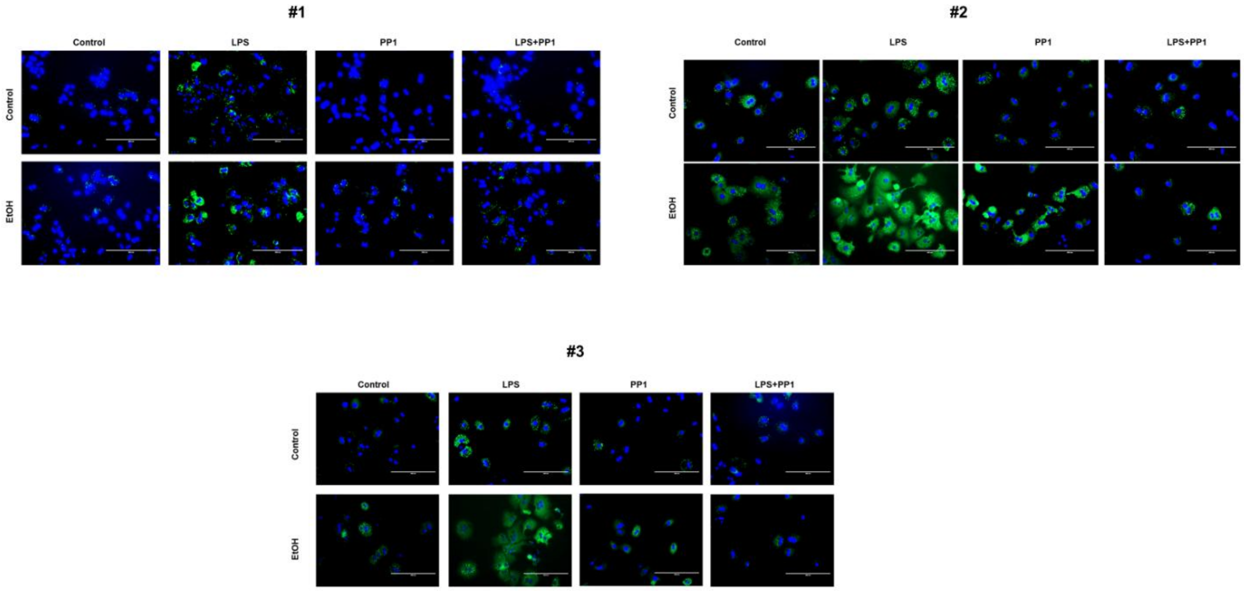

Figure 5F

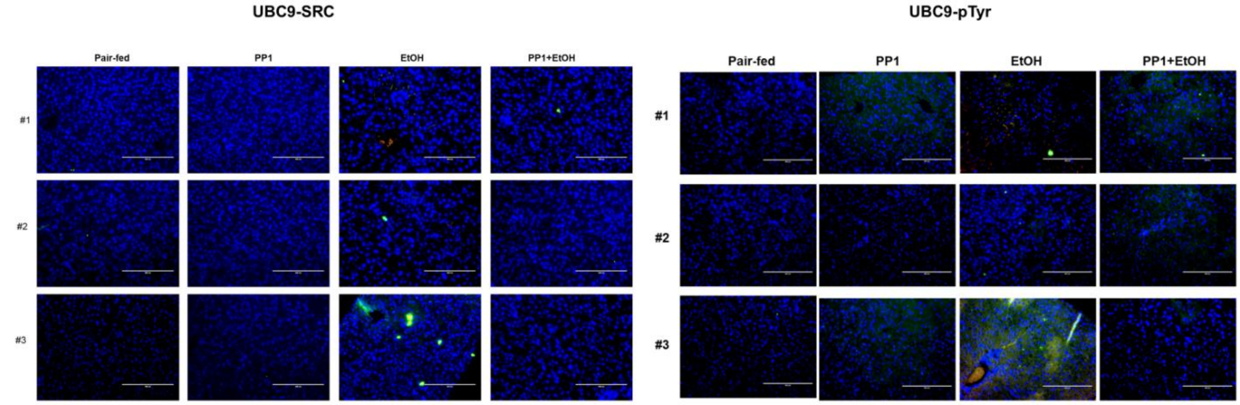

Figure 6A

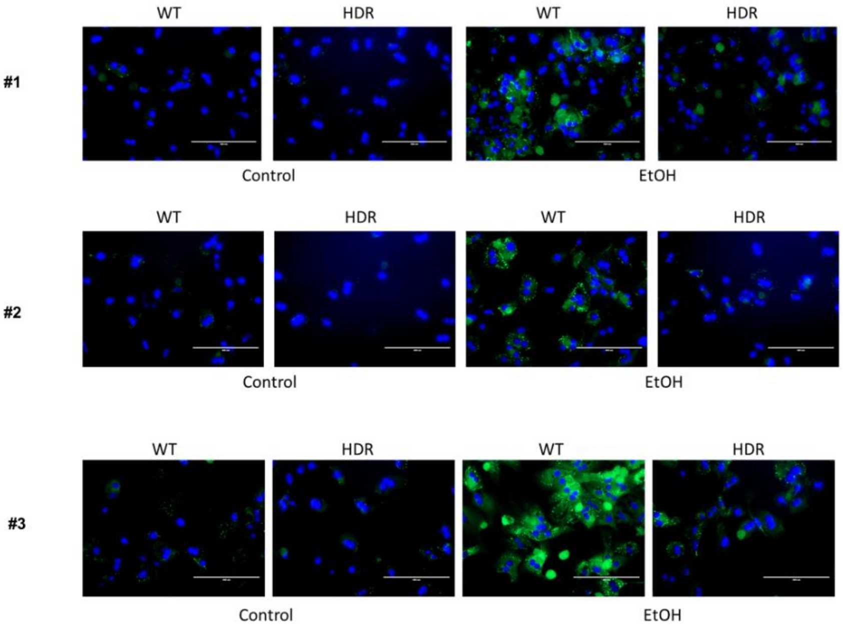

Figure 6D

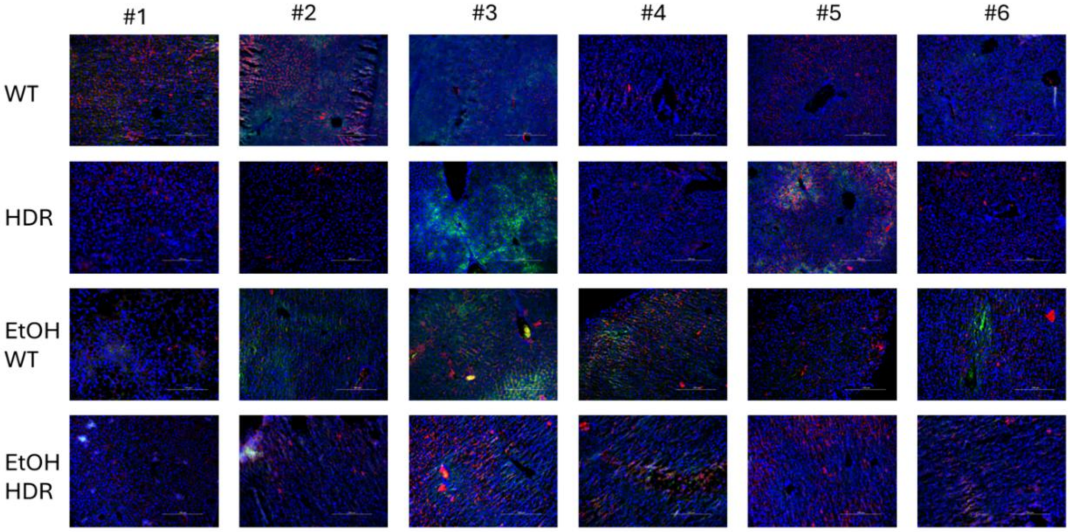

Figure 6F

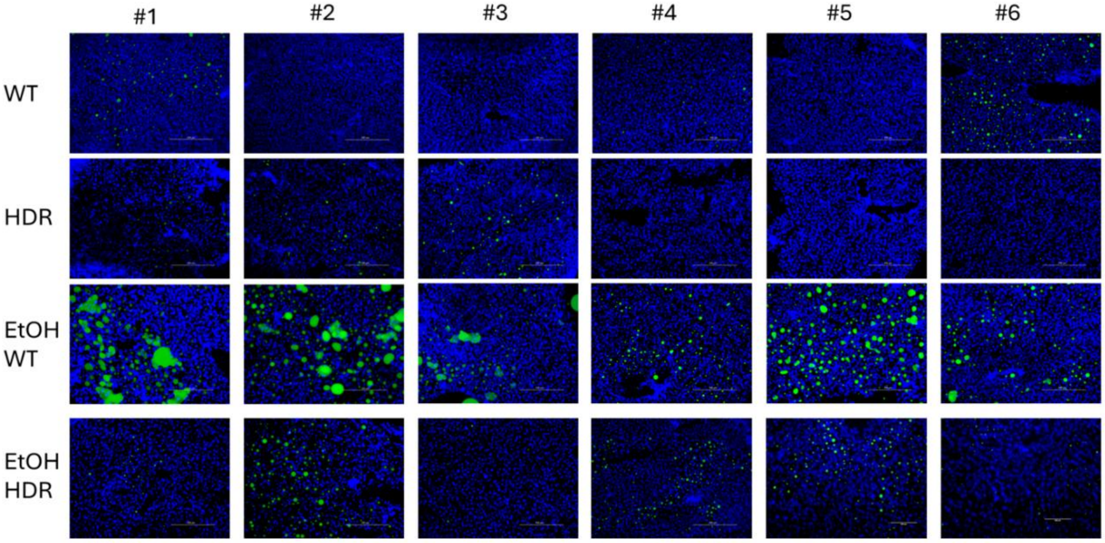

Figure 7B

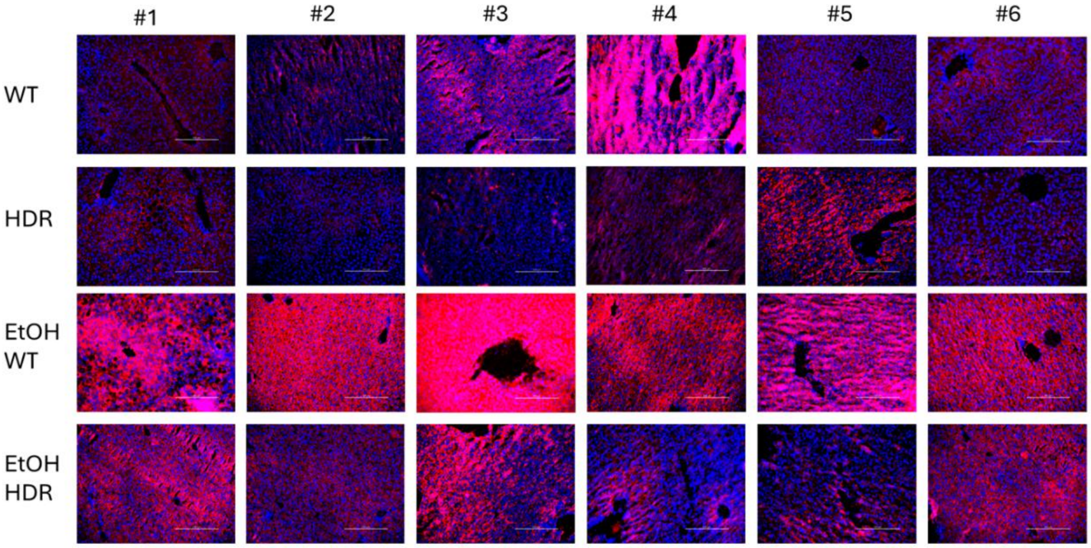

Figure 7C

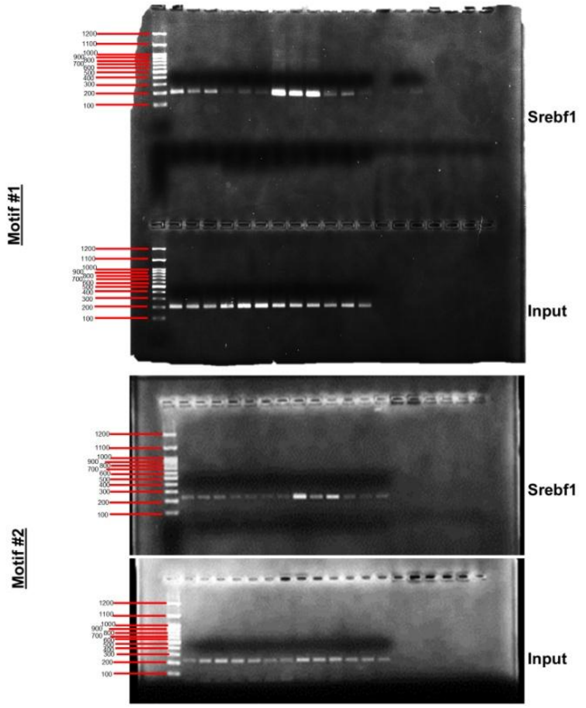

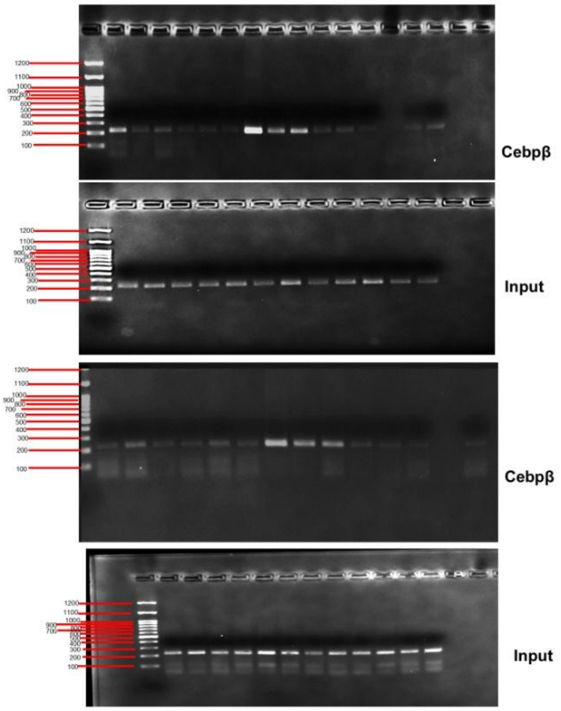

Figure S2

C

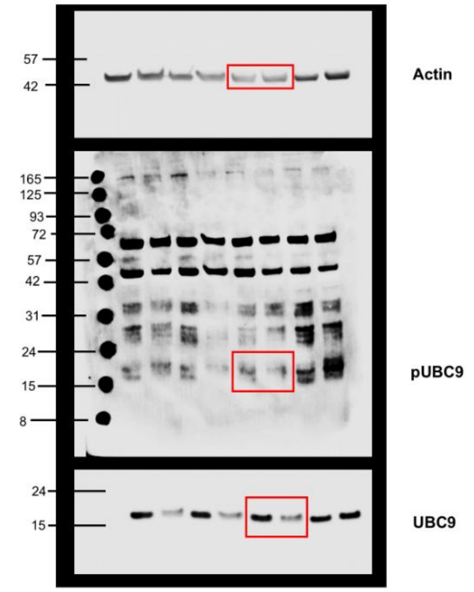

REFERENCES

1. J. Manthey, K. D. Shield, M. Rylett, O. S. M. Hasan, C. Probst, J. Rehm, Global alcohol exposure between 1990 and 2017 and forecasts until 2030: A modelling study. *Lancet* **393**, 2493–2502 (2019).

2. F. Åberg, Z. G. Jiang, H. Cortez-Pinto, V. Männistö, Alcohol-associated liver disease—Global epidemiology. *Hepatology* **80**, 1307–1322 (2024).

3. R. Celli, X. Zhang, Pathology of alcoholic liver disease. *J. Clin. Transl. Hepatol.* **2**, 103–109 (2014).

4. M. Fujimoto, M. Uemura, Y. Nakatani, K. Hoppo, T. Tamagawa, H. Kitano, M. Kikukawa, T. Ann, Y. Ishii, H. Kojima, S. Sakurai, R. Tanaka, T. Namisaki, R. Noguchi, T. Higashino, E. Kikuchi, K. Nishimura, A. Takaya, H. Fukui, Plasma endotoxin and serum cytokine levels in patients with alcoholic hepatitis: Relation to severity of liver disturbance. *Alcohol. Clin. Exp. Res.* **24**, 48S–54S (2000).

5. R. Kishore, M. R. McMullen, E. Cocuzzi, L. E. Nagy, Lipopolysaccharide-mediated signal transduction: Stabilization of TNF-alpha mRNA contributes to increased lipopolysaccharide-stimulated TNF-alpha production by Kupffer cells after chronic ethanol feeding. *Comp. Hepatol.* **3**, S31 (2004).

6. A. J. Nowak, B. Relja, The impact of acute or chronic alcohol intake on the NF-κB signaling pathway in alcohol-related liver disease. *Int. J. Mol. Sci.* **21**, 9407 (2020).

7. J. F. Lawler Jr, M. Yin, A. M. Diehl, E. Roberts, S. Chatterjee, Tumor necrosis factor-α stimulates the maturation of sterol regulatory element binding protein-1 in human hepatocytes through the action of neutral sphingomyelinase. *J. Biol. Chem.* **273**, 5053–5059 (1998).

8. Y. Kanamori, M. Murakami, M. Sugiyama, O. Hashimoto, T. Matsui, M. Funaba, Interleukin-1β (IL-1β) transcriptionally activates hepcidin by inducing CCAAT enhancer-binding protein δ (C/EBPδ) expression in hepatocytes. *J. Biol. Chem.* **292**, 10275–10287 (2017).

9. H. Shen, S. Liangpunsakul, Y. Iwakiri, G. Szabo, H. Wang, Immunological mechanisms and emerging therapeutic targets in alcohol-associated liver disease. *Cell. Mol. Immunol.* **22**, 1190–1204 (2025).

10. M. L. Tomasi, K. Ramani, M. Ryoo, Ubiquitin-conjugating enzyme 9 phosphorylation as a novel mechanism for potentiation of the inflammatory response. *Am. J. Pathol.* **186**, 2326–2336 (2016).

11. S. Prasad, P. K. Pal, SUMOylation: One small modification for proteins, multiple giant problems for mankind. *Mov. Disord.* **33**, 403 (2018).

12. M. L. Tomasi, I. Tomasi, K. Ramani, R. M. Pascale, J. Xu, P. Giordano, J. M. Mato, S. C. Lu, *S*-adenosyl methionine regulates ubiquitin-conjugating enzyme 9 protein expression and sumoylation in murine liver and human cancers. *Hepatology* **56**, 982–993 (2012).

13. F. Aillet, F. Lopitz-Otsoa, I. Egaña, R. Hjerpe, P. Fraser, R. T. Hay, M. S. Rodriguez, V. Lang, Heterologous SUMO-2/3-ubiquitin chains optimize IκBα degradation and NF-κB activity. *PLOS ONE* **7**, e51672 (2012).

14. J. M. Desterro, M. S. Rodriguez, R. T. Hay, SUMO-1 modification of IκBα inhibits NF-κB activation. *Mol. Cell* **2**, 233–239 (1998).

15. A. R. Poh, M. Ernst, Functional roles of SRC signaling in pancreatic cancer: Recent insights provide novel therapeutic opportunities. *Oncogene* **42**, 1786–1801 (2023).

16. L. J. Nelson, H. J. Wright, N. B. Dinh, K. D. Nguyen, O. V. Razorenova, F. S. Heinemann, Src kinase is biphosphorylated at Y416/Y527 and activates the CUB-domain containing protein 1/protein kinase C δ pathway in a subset of triple-negative breast cancers. *Am. J. Pathol.* **190**, 484–502 (2020).

17. I. Yakymovych, M. Yakymovych, A. Hamidi, M. Landström, C.-H. Heldin, The type II TGF-β receptor phosphorylates Tyr182 in the type I receptor to activate downstream Src signaling. *Sci. Signal.* **15**, eabp9521 (2022).

18. S. E. Byeon, Y.-S. Yi, J. Oh, B. C. Yoo, S. Hong, J. Y. Cho, The role of Src kinase in macrophage-mediated inflammatory responses. *Mediators Inflamm.* **2012**, 512926 (2012).

19. D. W. Kurniawan, R. Booijink, A. K. Jajoriya, G. Dhawan, D. Mishra, D. Oosterhuis, J. Argemi, G. Storm, P. Olinga, R. Bataller, S. K. Mohanty, D. P. Mishra, J. Prakash, R. Bansal, Src kinase as a potential therapeutic target in non-alcoholic and alcoholic steatohepatitis. *Clin. Transl. Discov.* **2**, e18 (2022).

20. M. D. Wheeler, H. Kono, M. Yin, M. Nakagami, T. Uesugi, G. E. Arteel, E. Gäbele, I. Rusyn, S. Yamashina, M. Froh, Y. Adachi, Y. Iimuro, B. U. Bradford, O. M. Smutney, H. D. Connor, R. P. Mason, S. M. Goyert, J. M. Peters, F. J. Gonzalez, R. J. Samulski, R. G. Thurman, The role of Kupffer cell oxidant production in early ethanol-induced liver disease. *Free. Radic. Biol. Med.* **31**, 1544–1549 (2001).

21. M. L. Tomasi, K. Ramani, M. Ryoo, C. Cossu, A. Floris, B. J. Murray, A. Iglesias-Ara, Y. Spissu, N. Mavila, SUMOylation regulates cytochrome P450 2E1 expression and activity in alcoholic liver disease. *FASEB J.* **32**, 3278–3288 (2018).

22. K. Alexandropoulos, G. Cheng, D. Baltimore, Proline-rich sequences that bind to Src homology 3 domains with individual specificities. *Proc. Natl. Acad. Sci. U.S.A.* **92**, 3110–3114 (1995).

23. U. Dionne, É. Bourgault, A. K. Dube, D. Bradley, F. J. M. Chartier, R. Dandage, S. Dibyachintan, P. C. Després, G. D. Gish, N. T. H. Pham, M. Létourneau, J.-P. Lambert, N. Doucet, N. Bisson, C. R. Landry, Protein context shapes the specificity of SH3 domain-mediated interactions in vivo. *Nat. Commun.* **12**, 1597 (2021).

24. Z. Wang, P. A. Cole, Catalytic mechanisms and regulation of protein kinases. *Methods Enzymol.* **548**, 1–21 (2014).

25. T. H. Leu, S. Charoenfuprasert, C. K. Yen, C. W. Fan, M. C. Maa, Lipopolysaccharide-induced c-Src expression plays a role in nitric oxide and TNF-α secretion in macrophages. *Mol. Immunol.* **43**, 308–316 (2006).

26. J. L. Kang, H. J. Jung, K. Lee, H. R. Kim, Src tyrosine kinases mediate crystalline silica-induced NF-κB activation through tyrosine phosphorylation of IκB-α and p65 NF-κB in RAW 264.7 macrophages. *Toxicol. Sci.* **90**, 470–477 (2006).

27. F. Wang, W. Zhang, C. Wang, X. Fang, H. Cheng, S. Liu, X. L. Chen, Inhibitor of Tec kinase, LFM-A13, decreases pro-inflammatory mediators production in LPS-stimulated RAW264.7 macrophages via NF-κB pathway. *Oncotarget* **8**, 34099–34110 (2017).

28. W. Yi-Bin, L. Xiang, Y. Bing, Z. Qi, J. Fei-Tong, W. Minghong, Z. Xiangxiang, K. Le, L. Yan, S. Ping, G. Yufei, X. Ye, W. Chun-Yan, Inhibition of the CEBPβ-NFκB interaction by nanocarrier-packaged Carnosic acid ameliorates glia-mediated neuroinflammation and improves cognitive function in an Alzheimer's disease model. *Cell Death Dis.* **13**, 318 (2022).

29. J. Ma, C. Liu, Y. Yang, J. Yu, J. Yang, S. Yu, J. Zhang, L. Huang, C/EBPβ acts upstream of NF-κB P65 subunit in Ox-LDL-induced IL-1β production by macrophages. *Cell. Physiol. Biochem.* **48**, 1605–1615 (2018).

30. G. He, M. Karin, NF-κB and STAT3 – Key players in liver inflammation and cancer. *Cell Res.* **21**, 159–168 (2011).

31. H. Lu, X. Lei, Q. Zhang, Moderate activation of IKK2-NF-kB in unstressed adult mouse liver induces cytoprotective genes and lipogenesis without apparent signs of inflammation or fibrosis. *BMC Gastroenterol.* **15**, 94 (2015).

32. J. Liu, M. Sha, Q. Wang, Y. Ma, X. Geng, Y. Gao, L. Feng, Y. Shen, Y. Shen, Small ubiquitin-related modifier 2/3 interacts with p65 and stabilizes it in the cytoplasm in HBV-associated hepatocellular carcinoma. *BMC Cancer* **15**, 675 (2015).

33. Z. Liu, Z. Wang, J. Wang, S. Xu, T. Zhang, Uridine, a therapeutic nucleoside, exacerbates alcoholic liver disease via SRC kinase activation: A network toxicology and molecular dynamics perspective. *Int. J. Mol. Sci.* **26**, 5473 (2025).

34. M. Sharma, V. Gadang, A. Jaeschke, Critical role for mixed-lineage kinase 3 in acetaminophen-induced hepatotoxicity. *Mol. Pharmacol.* **82**, 1001–1007 (2012).

35. J. F. Lawler Jr, M. Yin, A. M. Diehl, E. Roberts, S. Chatterjee, Tumor necrosis factor-alpha stimulates the maturation of sterol regulatory element binding protein-1 in human hepatocytes through the action of neutral sphingomyelinase. *J. Biol. Chem.* **273**, 5053–9 (1998).

36. S. Z. Lin, Y. Xie, Y. Q. Cheng, R. Xue, Y. S. Su, M. Liu, Y. W. Chen, J. G. Fan, C/EBPβ-VCAM1 axis in Kupffer cells promotes hepatic inflammation in MASLD. *JHEP Rep.* **7**, 101418 (2025).

37. M. Schonfeld, K. Nataraj, S. Weinman, I. Tikhanovich, C/EBPβ transcription factor promotes alcohol-induced liver fibrosis in males via HDL remodeling. *Hepatol. Commun.* **9**, e0645 (2025).

38. R. A. Ansari, K. Husain, S. A. Rizvi, Role of transcription factors in steatohepatitis and hypertension after ethanol: The epicenter of metabolism. *Biomolecules* **6**, 29 (2016).

39. M. G. Neuman, Y. Maor, R. M. Nanau, E. Melzer, H. Mell, M. Opris, L. Cohen, S. Malnick, Alcoholic liver disease: Role of cytokines. *Biomolecules* **5**, 2023–2034 (2015).

40. A. Bertola, S. Mathews, S. H. Ki, H. Wang, B. Gao, Mouse model of chronic and binge ethanol feeding (the NIAAA model). *Nat. Protoc.* **8**, 627–637 (2013).

41. E. A. Sneddon, R. D. White, A. K. Radke, Sex differences in binge-like and aversion-resistant alcohol drinking in C57BL/6J mice. *Alcohol. Clin. Exp. Res.* **43**, 243–249 (2019).
